# Correlation between Circulating Fungal Biomarkers and Clinical Outcome in Invasive Aspergillosis

**DOI:** 10.1371/journal.pone.0129022

**Published:** 2015-06-24

**Authors:** Dionysios Neofytos, Radha Railkar, Kathleen M. Mullane, David N. Fredricks, Bruno Granwehr, Kieren A. Marr, Nikolaos G. Almyroudis, Dimitrios P. Kontoyiannis, Johan Maertens, Rebecca Fox, Cameron Douglas, Robert Iannone, Eunkyung Kauh, Norah Shire

**Affiliations:** 1 Department of Medicine, Johns Hopkins University, Baltimore, MD, United States of America; 2 Department of Medicine, Memorial Sloan-Kettering Cancer Center, New York, NY, United States of America; 3 Clinical Research, Merck & Co., Inc., Kenilworth, NJ, United States of America; 4 Department of Medicine, University of Chicago, Chicago, IL, United States of America; 5 Division of Allergy and Infectious Diseases, Fred Hutchinson Center, Seattle, WA, United States of America; 6 Division of Infectious Diseases, University of Texas M.D. Anderson Cancer Center, Houston, TX, United States of America; 7 Roswell Park Cancer Institute, State University of New York, Buffalo, NY, United States of America; 8 Department of Haematology, University Hospital Gasthuisberg, Leuven, Belgium; Leibniz Institute for Natural Products Research and Infection Biology- Hans Knoell Institute, GERMANY

## Abstract

Objective means are needed to predict and assess clinical response in patients treated for invasive aspergillosis (IA). We examined whether early changes in serum galactomannan (GM) and/or β-D-glucan (BDG) can predict clinical outcomes. Patients with proven or probable IA were prospectively enrolled, and serial GM and BDG levels and GM optical density indices (GMI) were calculated twice weekly for 6 weeks following initiation of standard-of-care antifungal therapy. Changes in these biomarkers during the first 2 and 6 weeks of treatment were analyzed for associations with clinical response and survival at weeks 6 and 12. Among 47 patients with IA, 53.2% (25/47) and 65.9% (27/41) had clinical response by weeks 6 and 12, respectively. Changes in biomarkers during the first 2 weeks were associated with clinical response at 6 weeks (GMI, *P* = 0.03) and 12 weeks (GM+BDG composite, *P* = 0.05; GM, *P* = 0.04; GMI, *P* = 0.02). Changes in biomarkers during the first 6 weeks were also associated with clinical response at 6 weeks (GM, *P* = 0.05; GMI, *P* = 0.03) and 12 weeks (BDG+GM, *P* = 0.02; GM, *P* = 0.02; GMI, *P* = 0.01). Overall survival rates at 6 weeks and 12 weeks were 87.2% (41/47) and 79.1% (34/43), respectively. Decreasing biomarkers in the first 2 weeks were associated with survival at 6 weeks (BDG+GM, *P* = 0.03; BDG, *P* = 0.01; GM, *P* = 0.03) and at 12 weeks (BDG+GM, *P* = 0.01; BDG, *P* = 0.03; GM, *P* = 0.01; GMI, *P* = 0.007). Similar correlations occurred for biomarkers measured over 6 weeks. Patients with negative baseline GMI and/or persistently negative GMI during the first 2 weeks were more likely to have CR and survival. These results suggest that changes of biomarkers may be informative to predict and/or assess response to therapy and survival in patients treated for IA.

## Introduction

Invasive fungal infections (IFI) continue to pose a significant challenge in the management of immunocompromised patients with relatively high mortality rates [1–3]. A composite endpoint of clinical, radiologic and microbiologic outcomes has historically been used to assess clinical response of patients with invasive aspergillosis (IA) to therapy [4, 5]. Since response criteria often rely on subjective assessments, ambiguities are frequent. Objective assessments based on evidence of radiologic or microbiologic improvement also have significant limitations. Radiologic lesions followed by serial imaging (at times difficult to obtain) may initially increase in size as a function of the natural course of infection or immune reconstitution rather than reflecting poor treatment response [6]. Additionally, the sensitivity of conventional microbiologic diagnostics, even in bronchoalveolar lavage (BAL) specimens for IA, is suboptimal and serial microbiologic assessments are impractical if an invasive procedure is required [7, 8]. Robust biomarkers of IFIs that can reliably predict clinical outcome, particularly early in the course of therapy, can represent a valuable tool in guiding antifungal treatment in clinical practice and antifungal drug development [9, 10].

Galactomannan (GM) is a polysaccharide cell-wall component, released during *Aspergillus* species hyphal growth. A double-sandwich enzyme-linked immunosorbent assay (ELISA) has been approved by the US Food and Drug Administration for diagnostic use with serum and BAL samples (Platelia *Aspergillus* assay, Bio-Rad Laboratories, Hercules, CA). (1,3)-β-D-glucan (BDG) is a cell wall component of many fungi, capable of activating factor G of the horseshoe crab coagulation cascade and can be detected with a chromogenic substrate. The Fungitell β-glucan assay (Associates of Cape Cod, Cape Cod, MA), although highly sensitive, is not specific for *Aspergillus* [11].

Despite the growing body of literature on diagnostics, few studies have examined the prognostic value of these two fungal biomarkers utilized together [12–21]. We hypothesized that standardised sampling and a rigorous statistical approach combining these biomarkers would provide additional value in predicting patient prognosis and response to antifungal therapy. Therefore, we assessed the correlation between GM and BDG levels early in treatment and 6- and 12-week clinical outcomes in patients with IA.

## Methods

### Study Design

This was a prospective, non-interventional study (Protocol 089 (S1 File)) conducted at 18 centers across the US and Belgium. Patients were eligible for enrollment if they had (i) possible, probable, or proven IA per the revised 2008 criteria developed by the European Organization for Research and Treatment of Cancer/Invasive Fungal Infections Co-Operative Group (EORTC/IFICG) and the National Institute of Allergy and Infectious Diseases—Mycoses Study Group (NIAID-MSG) [22], (ii) a baseline blood sample for biomarker analysis within 24 hours of antifungal therapy initiation, and (iii) a baseline imaging study within 72 hours before or 24 hours following treatment initiation. Patients with possible IA were excluded from the study if a probable or proven diagnosis was not established by day (D) 14. Respiratory sources for mycological criteria assessment included bronchoalveolar lavage (BAL), sputum, brochial brushings, or sinus aspirate, per local standard of care. Patients were considered evaluable if (by D14) they were alive and had (1) proven or probable IA, (2) at least 2 valid results for each biomarker, and (3) available clinical outcome determination. Patients were prospectively followed for at least 12 weeks following D1, defined as the first day of systemic antifungal therapy administration. Antifungal treatment was administered per local standard-of-care. Patients on experimental therapies were permitted to enroll.

### Ethics Statement

This prospective study was approved by the Institutional Review Board (IRB) or Ethics Committee of each institution: the IRB of the University of Michigan Medical School; the University of Chicago Division of Biological Sciences and University of Chicago Medical Center IRB; the IRB at Roswell Park Cancer Institute; Stanford IRB; Wayne State University IRB; Fred Hutchinson Cancer Research Center IRB; the University of Texas M.D. Anderson Cancer Center IRB; Wake Forest University Health Sciences IRB; Summa Health System IRB; the University of Alabama at Birmingham Review Board for Human Use; the University of Minnesota IRB; the University of Arkansas for Medical Sciences IRB; the University of Wisconsin Health Sciences IRB; Johns Hopkins Medicine IRB; the University of Pennsylvania IRB; Henry Ford Health System IRB; Oregon Health & Science University IRB; and Universitair Ziekenhuis Leuven Medical Ethics Committee. Per the IRB-approved protocol, patients ≥16 years of age could enter the study; however, no one under the age of 18 was enrolled. Written informed consent was obtained from each patient before any study procedures were performed.

### Data Collection

Demographics, underlying disease, and IA-specific data, including mycological, radiologic and detailed treatment information were prospectively collected. Blood was collected twice per week during weeks 1–6 and weekly during weeks 7–12 for biomarker analysis. Serum GM and BDG were analyzed at a central laboratory to ensure analytic consistency for study endpoints. However, all centers performed local tests for labs that would be required for patient care, which may have included GM and BDG. Patients were assessed for adverse experiences associated with the study procedures through clinical evaluation by the study investigator as part of the patient’s standard-of-care. In cases where a patient died, pertinent autopsy information was collected if an autopsy was performed. Radiologic studies consistent with baseline method were conducted at 6 and 12 weeks.

### Assessment of Clinical Response

Patient outcomes were assessed at 6 and 12 weeks post-treatment initiation according to adjusted published definitions [5]. Briefly, complete or partial response was defined as survival and resolution or improvement, respectively, of all attributable clinical, radiologic, and microbiologic findings of infection. Failure was defined as survival and minor or no improvement or worsening of clinical, radiologic, and microbiologic findings of IA. Patients with complete or partial response were coded as responders (R) and those with failure as non-responders (NR). Patients who died prior to assessment were categorized as failures. Patients who discontinued the study for any reason and did not have a clinical outcome assessment prior to that time were considered non-evaluable and were excluded from the analysis. Response was assessed by an adjudication committee (two blinded clinicians and one radiologist). Consensus was reached if two of the three assessments matched. Instances of discordance were resolved by the committee independent of the study sponsor.

### Biomarkers

GM was measured using the Platelia *Aspergillus* assay (Bio-Rad Laboratories, Hercules, CA). Absorbance values were used to calculate the ng/mL GM, based on validated standards, so that GM could be analyzed on a continuous basis rather than as a dichotomous variable. This was done to explore results with a broad dynamic range and to maximize sensitivity to changes over time. To define the lower level of quantification (LLOQ), a patient serum sample with low GM value, near the limit of detection, was assayed in duplicate four times and had an optical density index (ODI) value > 0.1; the mean ODI was 0.194, which yielded a GM of 0.426 ng/mL using standard curve. Subtracting 2 standard deviations from this value gave 0.355 ng/mL as the LLOQ. The serum GM ODI (GMI) was determined as part of the *post-hoc* analysis, with a single value of ≥0.5 considered positive. BDG was measured using the Fungitell assay (Associates of Cape Cod, Cape Cod, MA) within a range from non-detectable (<31 pg/mL) to >500 pg/mL; for values >500 pg/mL, samples were diluted in reagent-grade water and re-tested to obtain accurate results [23]. A cutoff of 80 pg/mL was used to define positivity [24].

### Statistical Methods

The primary endpoint was prediction of 6-week clinical outcomes (R vs NR) using the average of the z-scores of the time-weighted averages of BDG and GM during the first 2 weeks of therapy. O’Brien’s global statistic was computed as the average of the z-scores of the time-weighted averages across the biomarkers, where the z-score for each patient for a given biomarker was computed by subtracting the mean and dividing by the standard deviation of the time-weighted averages [25]. A t-test was performed to evaluate the difference in the means of this endpoint between R and NR at week 6.

In exploratory analyses, we evaluated (i) the average of the z-scores of the time-weighted averages of BDG and GM from baseline to week 6, and (ii) the time-weighted averages separately for BDG, GM, and GMI, from baseline to weeks 2 and 6, and their association with clinical response and survival at weeks 6 and 12. *Post-hoc* multivariable logistic regression analyses assessed the following independent variables as possible predictors of clinical response and survival at weeks 6 and 12: age, gender, ethnicity (Caucasian vs other), underlying disease (hematologic malignancy vs other), prior corticosteroid use (yes/no), baseline GMI and BDG positivity, certainty of IA diagnosis (probable vs proven), primary antifungal therapy (voriconazole vs other), and initiation of antifungal therapy before vs after baseline biomarkers were obtained. Additionally, using GMI as a dichotomous variable (positive: ≥0.5 vs negative), we studied changes of GMI status during the first 2 weeks as a predictor of clinical response and survival at weeks 6 and 12. Finally, Kaplan-Meier survival curves with log-rank tests were produced to compare survival among (a) patients with hematologic malignancies or stem-cell transplant (SCT) vs other underlying diseases, and (b) patients with positive vs negative baseline GMI. *P*-values comparing the proportion of patients with a certain characteristic between groups were based on Fisher’s exact test. No multiplicity adjustments were made.

## Results

### Patient Characteristics

Fifty-one (44%) of 116 patients enrolled were eligible for evaluation by D14, based on a diagnosis of proven or probable IA. One patient was not evaluable at week 6, and three patients were determined to be possible IA cases, leaving a final analysis population of 47 patients, 9 with proven IA and 38 with probable IA (Table 1). A total of 669 (mean: 14.5 per patient; range: 3–19) BDG and 695 (mean: 15.1 per patient; range: 4–19) GM tests were performed. The overall means and ranges for BDG, GM, and GMI were 747.6 pg/mL (<31, 73950), 1.0 ng/mL (<0.355, 120.8), and 0.48 (0.04, 9.97), respectively. Twenty (42.6%) and 33 (70.2%) patients had a positive BDG at baseline and at some point during the study, respectively; 13 (27.7%) patients had undetectable BDG during the study, and one patient did not have BDG performed. Fourteen (29.8%) and 18 (38.3%) patients had positive GM at baseline and at some point during the study, respectively; 28 (59.6%) patients had undetectable GM throughout the study, and one patient did not have GM performed. Among 46 patients with available data, those with a haematologic malignancy were more likely to have a detectable GM (11/25, 44.0%) compared to others (3/21, 14.3%; *P* = 0.05). Baseline blood samples were obtained before treatment initiation in 24 (52.2%) patients; 8 (33.3%) of these 24 patients had detectable baseline GM compared to 6 of 22 (27.3%; *P* = 0.75) patients whose baseline blood samples were obtained after treatment initiation.

**Table 1:** Patient baseline characteristics.

	All Patients
	N = 47 (%)
**Demographics**	
Age, Median; Range (years)	60 (19–74)
Gender, Female	15 (31.9)
Ethnicity, Caucasian	40 (85.1)
**Underlying Disease** ^a^	
Hematologic malignancy	26 (56.3)
Stem cell transplant	13 (27.7)
Solid organ transplant	13 (27.7)
Solid tumor	4 (8.5)
Inherited severe immunodeficiency	1 (2.1)
**Other Comorbidities** ^a^	
Neutropenia (<500 cells/mm^3^)	15 (31.9)
Corticosteroids^b^	25 (53.2)
Graft-versus-host disease	4 (8.5)
Cytotoxic chemotherapy	14 (29.8)
**Certainty of Diagnosis of IA**	
Proven	9 (19.1)
Probable	38 (80.9)
**Microbiologic Diagnosis of IA** ^a^	
Biopsy	7 (14.9)
Positive culture for *Aspergillus* species^c^	19 (40.4)
Positive serum GMI^d^	28 (59.6)
Positive BDG^d^	6 (12.7)
**Site of IA** ^a^	
Lower respiratory tract^e^	44 (93.6)
Sinusitis	4 (8.5)
**Antifungal Treatment**	
Voriconazole	30 (63.8)
Amphotericin-B formulations^f^	8 (17.0)
Echinocandins	3 (6.4)
Other^g^	2 (4.3)
Combination therapy^h^	4 (8.5)

IA: Invasive Aspergillosis, GMI: Galactomannan Optical Density Index, BDG: β-D-glucan.

^a^Underlying disease, comorbidities, and microbiologic diagnostic tests were not mutually exclusive.

^b^Administration of corticosteroids (0.3 mg/kg/day of prednisone equivalent) for a minimum of 3 weeks prior to enrollment.

^c^Four patients with proven IA had a positive culture for *Aspergillus* species: 3 from normally sterile sites and 1 from a sputum/bronchoalveolar lavage culture.

^d^Patients with positive GM and BDG as tested at the institutions they were enrolled at are included in this Table. Some of them had negative GM and BDG when tested at the central laboratory for the purposes of this study.

^e^One patient had both sinusitis and lower tract respiratory involvement.

^f^Six and 2 patients were treated with liposomal amphotericin B and amphotericin B deoxycholate, respectively.

^g^One patient was treated with fluconazole alone and the other patient received blinded treatment with a mould acting agent as part of a clinical trial.

^h^Patients received more than one agent concomitantly: echinocandin and voriconazole (n = 2), echinocandin and amphotericin B deoxycholate (n = 1), and voriconazole and amphotericin B deoxycholate (n = 1).

### Clinical Response

Twenty-five (53.2%) patients were assessed to have complete or partial response (R) and 22 (46.8%) as failure (NR) at week 6. Baseline GMI was positive in 3 (12%) R vs 8 (36.4%) NR (*P* = 0.08). Excluding 6 non-evaluable patients at week 12 (3 discontinued the study, 1 had moved, and 2 had missing response assessment), 27 (65.9%) and 14 (34.1%) of the remaining 41 patients were R and NR, respectively. Baseline GMI positivity differed between week-12 R (4, 14.8%) and NR (6, 42.9%; *P* = 0.06).

Changes in the mean z-scores for the time-weighted averages (means) of the composite of BDG+GM from baseline to week 2 of antifungal therapy (primary endpoint) did not predict the clinical response at week 6 (Table 2). However, there was a trend toward an association between changes from baseline to week 2 in the means of BDG+GM (*P* = 0.13) and GM (*P* = 0.06), and an association of GMI value (*P* = 0.03) with 6-week clinical response. 12-week clinical response was associated with a decline in the means of BDG+GM (*P* = 0.05), GM (*P* = 0.04), and GMI (*P* = 0.02) from baseline to week 2. Decreased means in biomarkers during the first 6 weeks of treatment were associated with week-6 clinical response for GM (*P* = 0.05) and GMI (*P* = 0.03) and week-12 clinical response for BDG+GM (*P* = 0.02), GM (*P* = 0.02), and GMI (*P* = 0.01).

**Table 2:** (a) Mean Z-scores for the time-weighted averages of galactomannan and beta-D-glucan composite values, and (b) time-weighted averages for beta-D-glucan values, galactomannan values, and galactomannan optical density index, in responders vs non-responders at week 6 and week 12.

	GM+BDG (mean z-score)		BDG^a^ (pg/mL)		GM^a^ (ng/mL)		GMI^a^	
Response	Baseline to W-2	Baseline to W-6	Baseline to W-2	Baseline to W-6	Baseline to W-2	Baseline to W-6	Baseline to W-2	Baseline to W-6
**Week 6**								
R, Mean (N)	-0.10 (25)	-0.12 (25)	929 (24)	693 (24)	0.48 (25)	0.29 (25)	0.27 (25)	0.18 (25)
NR, Mean (N)	0.24 (22)	0.31 (22)	2174 (20)	999 (20)	1.49 (20)	1.53 (21)	0.85 (20)	0.83 (21)
Mean Difference	0.34	0.43	1245	306	1.01	1.24	0.58	0.65
90% CI	-0.17, 0.84	-0.11, 0.97	-1825, 4315	-1080, 1694	-0.06, 2.09	-0.01, 2.49	0.09, 1.07	0.06, 1.23
***P* value**	**0.13**	**0.09**	**0.25**	**0.36**	**0.06**	**0.05**	**0.03**	**0.03**
**Week 12**								
R, Mean (N)	-0.12 (27)	-0.16 (27)	873 (26)	656 (26)	0.56 (25)	0.29 (26)	0.33 (25)	0.19 (25)
NR, Mean (N)	0.42 (14)	0.55 (14)	3555 (12)	1607 (12)	1.91 (14)	2.19 (14)	1.05 (14)	1.17 (14)
Mean Difference	0.54	0.71	2682	951	1.35	1.89	0.72	0.98
90% CI	-0.01, 1.10	0.12, 1.3	-1083, 6447	-752, 2655	0.09, 2.62	0.44, 3.35	0.13, 1.29	0.30, 1.66
***P* value**	**0.05**	**0.02**	**0.12**	**0.18**	**0.04**	**0.02**	**0.02**	**0.01**

GM: Galactomannan, BDG: Beta-D-Glucan, GMI: Galactomannan Optical Density Index, W: Week, R: Responder, NR: Non-responder, N: Number, CI: Confidence Interval.

^**a**^ Some patients did not have data available for both biomarkers throughout the study; therefore, the numbers of patients with BDG, GM and/or GMI at weeks 2 and 6 differ from the total number of patients in the BDG and GM column.

### Survival

**Fig 1:**
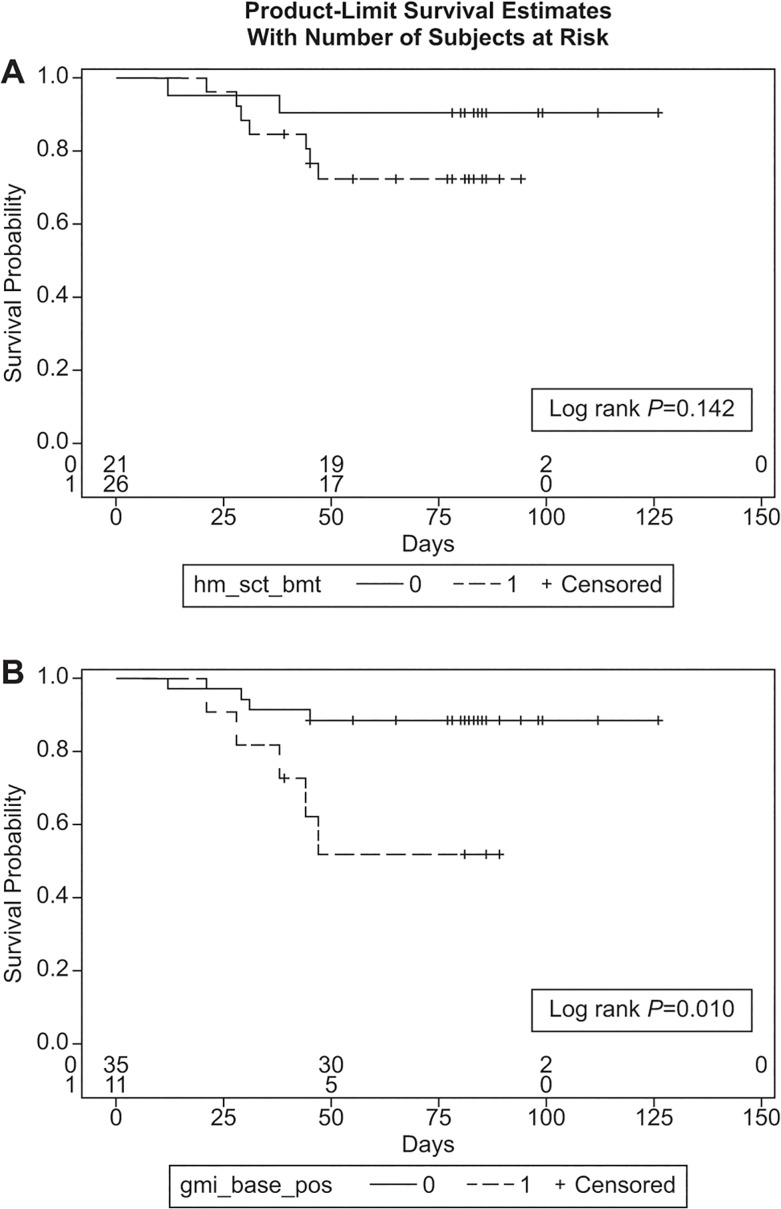
Kaplan-Meier survival curves among: (A) patients with hematologic malignancies and stem cell transplant recipients compared to patients with other underlying diseases, and (B) patients with positive vs negative baseline galactomannan enzyme immunoassay optical density index. In Fig 1A, 0 (solid line) represents “other populations” and 1 (dashed line) represents patients with “HSCT/BMT patients”. In Fig 1B, 0 (solid line) represents patients with negative GMI at baseline, and 1 (dashed line) represents patients with positive GMI at baseline.

Overall 6-week survival was 87.2%. Baseline GMI was positive in 8 (19.5%) of 41 patients who were alive vs 3 (50%) of 6 patients who had died by week 6 (*P* = 0.13). Excluding 4 patients (3 who discontinued the study and 1 who moved), 34 (79.1%) of 43 patients were alive by week 12. Survival did not significantly differ when patients with hematologic malignancy and SCT recipients were compared to all others (log-rank *P* = 0.14; Fig 1A). In contrast, patients with positive baseline GMI were more likely to have died by week 12 compared to those with negative baseline GMI (log-rank *P* = 0.009; Fig 1B). Survival at 6 and 12 weeks was associated with a decline in the means of BDG+GM (*P* = 0.03), BDG (*P* = 0.01), GM (*P* = 0.03) and BDG+GM (*P* = 0.01), BDG (*P* = 0.03), GM (*P* = 0.01), and GMI (*P* = 0.007) during the first 2 weeks of treatment, respectively (Table 3). Changes during the first 6 weeks of treatment were associated with 6-week survival for BDG (*P* = 0.02) and 12-week survival for BDG+GM (*P* = 0.004), GM (*P* = 0.004), and GMI (*P* = 0.003).

**Table 3:** (a) Mean Z-scores for the time-weighted averages of galactomannan and beta-D-glucan composite values, and (b) time-weighted averages for beta-D-glucan values, galactomannan values, and galactomannan optical density index, by patient survival (alive vs dead) at week 6 and week 12.

	GM+BDG (mean z-score)		BDG^a^ (pg/mL)		GM^a^ (ng/mL)		GMI^a^	
Response	Baseline to W-2	Baseline to W-6	Baseline to W-2	Baseline to W-6	Baseline to W-2	Baseline to W-6	Baseline to W-2	Baseline to W-6
**Week 6**								
A, Mean (N)	-0.05 (41)	0.01 (41)	675.7 (38)	512.3 (38)	0.7 (39)	0.7 (40)	0.45 (39)	0.43 (40)
D, Mean (N)	0.80 (6)	0.51 (6)	6685.8 (6)	2859.7 (6)	2.5 (6)	1.6 (6)	1.01 (6)	0.81 (6)
Mean Difference	0.85	0.50	6010.1	2347.4	1.8	0.9	0.56	0.39
90% CI	0.12, 1.58	-0.32, 1.31	1811, 10209	425, 4269	0.3, 3.4	-1.1, 2.7	-0.19, 1.29	-0.51, 1.29
***P* value**	**0.03**	**0.16**	**0.01**	**0.02**	**0.03**	**0.24**	**0.11**	**0.23**
**Week 12**								
A, Mean (N)	-0.11 (34)	-0.14 (34)	745.2 (32)	570.6 (32)	0.6 (32)	0.4 (33)	0.36 (32)	0.24 (33)
D, Mean (N)	0.72 (9)	0.92 (9)	5220.4 (8)	2276.5 (8)	2.5 (9)	3.0 (9)	1.31 (9)	1.50 (9)
Mean Difference	0.83	1.06	4475.2	1705.5	1.9	2.6	0.95	1.26
90% CI	0.22, 1.45	0.41, 1.71	421, 8529	140, 3551	0.6, 3.3	1.1, 4.2	0.33, 1.58	0.52, 2.0
***P* value**	**0.01**	**0.004**	**0.03**	**0.06**	**0.01**	**0.004**	**0.007**	**0.003**

GM: Galactomannan, BDG: Beta-D-Glucan, GMI: Galactomannan Optical Density Index, W: Week, A: Alive, D: Dead, N: Number, CI: Confidence Interval.

^**a**^ Some patients did not have data available for both biomarkers throughout the study; therefore, the numbers of patients with BDG, GM and/or GMI at weeks 2 and 6 differ from the total number of patients in the BDG and GM column.

### Outcome Predictors

In univariate analyses, a negative baseline GMI (<0.5) predicted a favorable 6-week clinical response (odds ratio, OR: 4.5, 95% CI, 1.09, 23.52, *P* = 0.05) and survival by 12 weeks (OR: 7.0, 95% CI, 1.42, 38.74, *P* = 0.02). Univariate analyses for all other independent variables and multivariable logistic regression analyses failed to identify any additional significant predictors of 6- and 12-week clinical response or survival.

Using GMI as a dichotomous variable (positive, ≥0.5, or negative) we assessed 6- and 12-week outcomes between patients whose baseline GMI remained positive during the first 2 weeks (+/+) to those whose baseline GMI turned negative from positive (+/-), those who turned positive from negative (-/+), or those who remained negative (-/-) by week 2 (Table 4). It should be noted that only one patient was in the (+/-) group, so no analytical results could be generated for this category. Patients with negative GMI during the first 2 weeks of treatment were more likely to have a favorable clinical response and be alive by week 12, when compared to patients with consistently positive GMI.

**Table 4:** Association of galactomannan assay optical density index (GMI) as a dichotomous variable (positive [GMI ≥ 0.5] vs negative) between baseline and week 2 of treatment with clinical response and survival at 6 and 12 weeks.

	GMI	OR (95% CI)	*P*-value
	(Baseline / Week 2)		
**Clinical Response** ^**a**^ ^,^ ^b^			
Week 6	-/- vs +/+	4.1 (0.9, 18.8)	0.07
	-/+ vs +/+	2.3 (0.1, 51.0)	0.6
Week 12	-/- vs +/+	4.5 (1.0, 20.7)	0.05
	-/+ vs +/+	1.5 (0.07, 31.6)	0.8
**Survival**			
Week 6	-/- vs +/+	4.3 (0.7, 25.9)	0.1
	-/+ vs +/+	^c^	
Week 12	-/- vs +/+	6.5 (1.3, 33.0)	0.02
	-/+ vs +/+	^c^	

GMI: Galactomannan Optical Density Index, OR: Odds Ratio, CI: Confidence Interval

^a^ Clinical response: complete or partial response, as described in Methods [5].

^b^ Only one patient had positive GMI at baseline and negative GMI by week 2, so this category (+/-) was not included in the analysis.

^c^ Due to low number of observations, there was lack of model convergence and no results were generated.

## Discussion

This prospective multicenter study assessed the utility of serial serum biomarkers as predictors of response to treatment in immunocompromised patients with proven or probable IA. Data demonstrate that decreasing GMI titers during the first 2 weeks of IA treatment can predict favorable clinical responses at 6 and 12 weeks after initiation of therapy. In addition, a decline in BDG and GMI during the first 2 weeks of treatment is associated with survival at 12 weeks. Finally, we observed correlations between declining BDG and GMI levels during the first 6 weeks of treatment and clinical responses and survival.

Multiple studies have emphasized the utility of serum GMI kinetics in predicting clinical outcomes in hematologic malignancy patients and/or SCT recipients treated for IA [15, 19, 20, 26]. A recent meta-analysis showed a strong correlation between serum GMI (≤1 week before outcome assessment) with clinical response outcomes and survival [15]. Baseline negative GMI and/or changes in serum GMI during the first week of treatment have also been reported to predict improved outcomes in patients with IA [16, 19–21, 27]. These data are further supported by our findings, which demonstrate the novel observation that people who had negative or declining GMI in the first two weeks of therapy were more likely to be alive after 12 weeks. The proportion of people who survived in this cohort was relatively high compared with prior studies that have reported outcomes predominately on patients with hematologic malignancies and/or SCT recipients [2–4, 28]. Whether this is a reflection of underlying host variables and/or therapy is unclear. However, this observation could instruct a potentially useful monitoring algorithm early after initiation of antifungal therapy. Notably, only a small minority of patients had a baseline positive GMI, most likely reflecting the variable sensitivity of the assay based on underlying disease and antifungal treatment initiation [29, 30].

There are limited data describing changes in serum BDG titers in patients treated for IFIs [12, 17, 31–34]. It appears that BDG kinetic changes occur slowly (e.g. in ≥4–6 weeks after treatment initiation) in patients treated for a variety of IFIs, including IA [12, 17, 31, 32, 34]. Our findings are consistent with recent data that early changes in BDG kinetics do not correlate well with clinical response in patients with IA [17]. However, we observed that a decline in BDG during the first 2 weeks of treatment was associated with 6- and 12-week survival. These are the first data to suggest that BDG can be useful to predict IA survival when used in the appropriate context. Our findings may differ from prior observations, in part, due to the wider range of quantification for BDG above the threshold of 500 pg/mL previously used [17].

Using clinical and radiological evaluations to validate antifungal compounds for the treatment of IA is subject to multiple biases and does not always correlate with survival [9, 10]. Hence, there is an urgent need for more objective, reliable, easily applicable and reproducible non-invasive tests that can be used as adjuncts to assess response. Our findings suggest that serial monitoring of carefully selected biomarkers may serve as surrogate clinical response endpoints. Caution needs to apply, as our (and prior) data do not answer pertinent questions: (i) should GMI and/or BDG be considered as surrogate endpoints, and if so should they be used as a continuous or dichotomous variable and at what frequency, (ii) what is the desired rate of biomarker change to predict outcomes, and (iii) on which patient populations should they be used (e.g. hematologic malignancy vs all, those positive at baseline only vs all). With the exception of the global IA combination treatment clinical trial, it is unlikely that more meaningful, prospective data to assess the utility of these biomarkers will be available in the near future [35]. Our experience with this study (initially designed in 2009, enrolling patients from 2010 to 2012, and coming to completion 2 years later) having encountered multiple logistical challenges that significantly hindered patient enrollment suggests that similar prospective efforts may not be a realistic possibility in the future.

One of the major limitations of this study is the small number of patients enrolled, despite multiple amendments to allow enrollment ±24 hours of antifungal therapy initiation and a ±7 day window to assess response. The former might have contributed to lower baseline biomarker titers in a subset of patients. However, when analyzed separately, there were no significant differences when comparing patients who had their baseline biomarkers drawn before and after treatment initiation. Notably, 28 (59.6%) patients had a positive baseline GMI reported by the local laboratory at their institution vs only 11 (23.4%) patients tested positive at baseline at the central laboratory. This discrepancy might have been due to including serum and/or BAL specimens locally vs only serum centrally, local reporting of positive screening GMI titers, which might have occurred prior to baseline testing and after administration of several doses of antifungal therapy, or sample processing and storage, although the latter was not likely based on the results of rigorous stability testing performed (data not shown). Although including a heterogeneous population of immunocompromised patients enhances the generalizability of our findings, it is likely that the observed trends in biomarkers could be more or less predictive in specific patient subpopulations. Finally, the use of GM concentration (as opposed to GMI) and the composite of GM and BDG may limit the applicability of these results for clinical practice purposes since these assay formats or combinations are not widely used.

## Conclusion

Our results suggest that monitoring GMI during the first 2 weeks of treatment may help inform patient progress and response to therapy, with declining levels indicating positive progress. In addition, 6-week GMI and BDG titers may be a useful tool to assess outcomes in the right context. Although larger studies are needed to validate these findings and to assess applicability in discreet patient populations, these findings support further exploration of the use of fungal biomarkers in clinical practice and for use as adjunctive measures of response in clinical trials.

## Supporting Information

S1 File Study Protocol No. 089: A Prospective, Non-Intervention, Observational Assessment of the Correlation Between Circulating Biomarkers of Fungal Bioburden and Clinical Outcome in the Setting of Invasive Aspergillosis. (PDF)
