# Supplementary material for: Correlation between Circulating Fungal Biomarkers and Clinical Outcome in Invasive Aspergillosis
Source: PLoS One. 2015 Jun 24;10(6):e0129022. doi: 10.1371/journal.pone.0129022 (PMC4480423; doi:10.1371/journal.pone.0129022)
Supplement: S1 File — (PDF) [file pone.0129022.s001.pdf]

Merck's policy on posting of redacted study protocols on journal websites is described in Merck Guidelines for Publication of Clinical Trials in the Scientific Literature on the www.merck.com website.

For publicly posted protocols, Merck redacts the background and rationale sections because these sections may contain proprietary information. Merck also redacts the names of any individuals due to privacy issues. The appendices generally are not provided because they may be lengthy and contain non-essential information. The publicly posted protocol includes all the key sections that are relevant to evaluating the study, specifically those sections describing the study objectives and hypotheses, the patient inclusion and exclusion criteria, the study design and procedures, the efficacy and safety measures, the statistical analysis plan, and amendments relating to those sections.

This report may include approved and non-approved uses, formulations, or treatment regimens. The results reported may not reflect the overall profile of a product. Before prescribing any product mentioned this report, healthcare professionals should consult local prescribing information for the product approved in their country.

Copyright © 2014 Merck Sharp & Dohme Corp., a subsidiary of Merck & Co., Inc.
All Rights Reserved. Not for regulatory or commercial use.

THIS PROTOCOL AND ALL OF THE INFORMATION RELATING TO IT ARE CONFIDENTIAL AND PROPRIETARY PROPERTY OF MERCK SHARP & DOHME CORP., A SUBSIDIARY OF MERCK & CO., INC., WHITEHOUSE STATION, NJ, U.S.A.

THIS PROTOCOL REPLACES THE ORIGINAL PROTOCOL AND ANY SUBSEQUENT AMENDMENTS AND SHOULD BE SIGNED BY ALL INVESTIGATORS SIGNING THE ORIGINAL PROTOCOL

SPONSOR:

Merck Sharp & Dohme Corp., a subsidiary of Merck & Co., Inc. (hereafter referred to as the **SPONSOR** or Merck)
One Merck Drive
P.O. Box 100
Whitehouse Station, NJ, 08889-0100, U.S.A.

Protocol-specific Sponsor Contact information can be found in the Administrative Binder.

TITLE:

A Prospective, Non-Intervention, Observational Assessment of the Correlation Between Circulating Biomarkers of Fungal Bioburden and Clinical Outcome in the Setting of Invasive Aspergillosis

INVESTIGATOR:

PRIMARY:

CLINICAL PHASE: Ib

US IND NUMBER: N/A

SITE:

INSTITUTIONAL REVIEW BOARD/ETHICS REVIEW COMMITTEE:

SUMMARY OF CHANGES

PRIMARY REASON FOR THIS AMENDMENT:

SECTION 2.7 and SECTION 3.5.9

Removal of the scheduled interim analysis from the study

OTHER CHANGES INCLUDED IN THE AMENDMENT:

SECTION 3.3.2 Adjudication Procedures

Added the following statement to clarify location of description of Independent Adjudication Board (IAB) operations:

Roles and responsibilities of the Independent Adjudication Board (IAB) and other operational procedures performed by the IAB, including guidelines for interpretation of radiographic images by the IAB, are outlined in a separate Charter.

SUMMARY OF CHANGES....2
PRIMARY REASON FOR THIS AMENDMENT: 2
OTHER CHANGES INCLUDED IN THE AMENDMENT:....2
1. SUMMARY....6
1.1 TITLE....6
1.2 INDICATION....6
1.3 SUMMARY OF RATIONALE....6
1.4 SUMMARY OF STUDY DESIGN....6
1.5 SAMPLE....7
1.6 DOSAGE/DOSAGE FORM, ROUTE, AND DOSE REGIMEN....7
1.7 STUDY FLOW CHART....8
2. CORE PROTOCOL....10
2.1 OBJECTIVES AND HYPOTHESES....10
2.1.1 Primary....10
2.1.2 Secondary....10
2.1.3 Exploratory Objectives....11
2.2 PATIENT INCLUSION CRITERIA....12
2.3 PATIENT EXCLUSION CRITERIA....13
2.4 STUDY DESIGN AND DURATION....13
2.4.1 Summary of Study Design....13
2.5 LIST OF MEASUREMENTS....14
2.5.1 Definitions of Outcome & Clinical Response....14
2.5.1.1 Successful Outcome: Complete Response (CR)....14
2.5.1.2 Successful Outcome: Partial Response (PR)....14
2.5.1.3 Outcome: Failure....15
2.5.2 Biomarkers....15
2.6 LIST OF SAFETY MEASUREMENTS....16
2.7 DATA ANALYSIS SUMMARY....16
3. PROTOCOL DETAILS....19
3.1 RATIONALE....19
3.1.1 Rationale for This Study....19
3.1.2 Rationale for Clinical Endpoints....20
3.1.3 Rationale for Choice of Study Population....21
3.1.4 Rationale for Assay Methods....21
3.1.4.1 (1,3)-β-D-glucan....21
3.1.4.2 Galactomannan....22
3.1.4.3 Fungal RNA....24
3.1.4.4 Serum archives....24

3.2 STUDY PROCEDURES 24
3.2.1 Informed Consent 24
3.2.1.1 General Informed Consent 24
3.2.2 Study Restrictions 25
3.2.2.1 Pregnancy & Contraception 25
3.2.3 Treatment 25
3.2.4 Procedures 25
3.2.4.1 Pre-Enrollment Period 26
3.2.4.2 Day 0 27
3.2.4.3 Weeks 1-5 27
3.2.4.4 Week 6 28
3.2.4.5 Weeks 7-11 28
3.2.4.6 Week 12 29
3.2.4.7 Follow-up 29
3.2.4.8 Assignment of Baseline Number/Screening 30
3.2.4.9 Allocation 30
3.2.4.10 Discontinuation/Withdrawal from Study 30
3.3 EFFICACY MEASUREMENTS 30
3.3.1 Clinical and Laboratory Measurements for Efficacy 30
3.3.1.1 Fungitell™ Assay 30
3.3.1.2 Galactomannan Assay 31
3.3.1.3 NASBA Assay 31
3.3.1.4 Anatomical Imaging Procedures 34
3.3.2 Adjudication Procedures 34
3.4 SAFETY MEASUREMENTS 35
3.4.1 Clinical and Laboratory Measurements for Safety 35
3.4.2 Recording Adverse Experiences 35
3.4.3 Reporting of Pregnancy to SPONSOR 36
3.4.4 Immediate Reporting of Adverse Experiences to the SPONSOR 36
3.4.4.1 Serious Adverse Experiences 36
3.4.5 Evaluating Adverse Experiences 36
3.4.6 SPONSOR Responsibility for Reporting Adverse Experiences 39
3.5 DATA ANALYSIS 39
3.5.1 Responsibility for Analyses 39
3.5.2 Hypotheses/Estimation 39
3.5.3 Analysis Endpoints 39
3.5.4 Analysis Populations 41
3.5.5 Statistical Methods 41
3.5.6 Multiplicity 43
3.5.7 Sample Size and Power Calculations 43
3.5.8 Subgroup Analyses and Effect of Baseline Factors 45
3.6 CLINICAL SUPPLIES 45

3.6.1 Clinical Supplies Disclosure 45
3.7 DATA MANAGEMENT 45
3.8 BIOLOGICAL SPECIMENS 45

4. ADMINISTRATIVE AND REGULATORY DETAILS 46

4.1 CONFIDENTIALITY 46
4.1.1 Confidentiality of Data 46
4.1.2 Confidentiality of Subject/Patient Records 46
4.1.3 Confidentiality of Investigator Information 46
4.2 COMPLIANCE WITH LAW, AUDIT, AND DEBARMENT 47
4.3 COMPLIANCE WITH FINANCIAL DISCLOSURE REQUIREMENTS 48
4.4 QUALITY CONTROL AND QUALITY ASSURANCE 49
4.5 COMPLIANCE WITH INFORMATION PROGRAM ON CLINICAL TRIALS FOR SERIOUS OR LIFE THREATENING CONDITIONS 49
4.6 PUBLICATIONS 49

5. LIST OF REFERENCES 50

6. APPENDICES 52

6.1 DEFINITION OF INVASIVE FUNGAL INFECTIONS 52
6.2 BLOOD VOLUME REQUIREMENTS 55

7. ATTACHMENTS 56

1. SUMMARY

1.1 TITLE

A Prospective, Non-Intervention, Observational Assessment of the Correlation Between Circulating Biomarkers of Fungal Bioburden and Clinical Outcome in the Setting of Invasive Aspergillosis

1.2 INDICATION

Not Applicable

1.3 SUMMARY OF RATIONALE

Redacted

1.4 SUMMARY OF STUDY DESIGN

This is a prospective, multi-center, non-intervention, observational study enrolling approximately 100 male and female patients ≥16 years of age with a presumptive diagnosis of possible, probable, or proven invasive Aspergillus infection as defined by the 2008 revised EORTC/MSG criteria to obtain laboratory and clinical data for at least 50 patients with proven or probable IA [1]. Patients will be enrolled in the study for approximately 12 weeks and will undergo regular, periodic blood sampling for evaluation of biomarkers indicative of aspergillus infection. Patients will be assessed for clinical response at both 6 and 12 weeks following initiation of anti-fungal therapy. Results of serum fungal biomarker levels measured in this study will not be available to the clinical

investigator for clinical care decisions. However, (1,3)-β-D-glucan and galactomannan levels can be obtained independently by the investigator as part of his/her standard clinical practice.

1.5 SAMPLE

Approximately 100 male and female patients ≥16 years of age with a presumptive diagnosis of possible, probable, or proven invasive Aspergillus infection as defined by EORTC/MSG criteria will be enrolled in the study. Additional patients may be enrolled to ensure a primary analysis population of at least 50 patients.

1.6 DOSAGE/DOSAGE FORM, ROUTE, AND DOSE REGIMEN

No study drug will be administered as part of this study. Patients will be treated for IA using standard-of-care methods at the discretion of the study investigator. The dose, route, regimen, and duration of standard of care therapy must be recorded in source documentation and on each patient's case report form.

1.7 STUDY FLOW CHART

	Pre-Enrollment Period[†]	Study Period					
Visit Number	V1	V2	V3				V4
		Day 0	Weeks 1-5 (Day 1-35)	Week 6 (Day 36-42)	Weeks 7-11 (Day 43-77)	Week 12 (Day 78-84)	Follow-up[‡‡‡]
Obtain Informed Consent	X						
Medical History and Prior Medication[‡]	X						
Review of Entry Criteria	X						
Physical Exam/Assessment of attributable symptoms[††††]	X	X		X		X	
Serum or urine β-hCG	X[¶¶¶]						
Initiation of systemic anti-fungal therapy[§]		X					
Day 14 Assessment			X[###]				
Vital Signs[‖]	X	X	X	X	X	X	
Blood Collection for Beta-Glucan and Galactomannan assays[¶]		X[#]	X[††]	X[††]	X[‡‡]	X[‡‡]	
Blood Collection for fungal RNA (NASBA) and fungal DNA analysis		X[#]	X[§§§]	X[‖‖‖]		X[‖‖‖]	
Blood Collection for serum archives and profiling assay[§§]		X[#]	X	X		X	
Review Concomitant Medications[‖‖]	X	X	X	X	X	X	X
Review Adverse Experiences[‖‖]		X	X	X	X	X	X
Clinical Response Assessment[¶¶]				X		X	
Anatomical Imaging Assessment	X[##]			X[†††]		X[†††]	

† Pre-enrollment procedures should be completed within 72 hours after patient is identified as potential for enrollment into study by the study investigator.

‡ Medical history must include the date of onset of signs and symptoms indicative of invasive Aspergillus infection. All antibiotic and antifungal medications taken prior to the initiation of anti-fungal therapy must be included in the source documentation and on the case report form. Documentation of initial diagnosis of IA including radiographs, microscopic analysis, culture results, non-culture based results and invasive procedures should be provided if available as relevant to eligibility criteria.

§ Initiation of systemic anti-fungal therapy defines beginning of Study Period, Day 0. Type and duration of anti-fungal therapy regimen is to be determined by the study investigator.

‖ Vital signs (oral temperature, BP, HR, RR) will be assessed prior to each blood collection throughout the study. Weight measurements will also be documented on the case report form.

¶ For all blood sample collections, usage of cotton-containing gauze, swabs, pads and bandages must be avoided. In addition, powder-free gloves must be worn by the phlebotomist. Powder-containing gloves must not be used to collect blood for biomarker assays.

# Baseline blood sample should be collected within 24 hours prior to initiation of anti-fungal therapy. If available, appropriately stored samples obtained by the study doctor or treating physician prior to the initiation of antifungal therapy may be used as the baseline sample for beta-glucan and galactomannan analysis if patient consents to the use of such a sample. If pre-therapy specimen cannot be obtained, baseline blood sample must be collected within 24 hours of initiation of systemic anti-fungal therapy.

†† Biomarkers specified in Section 2.5.2. Collections must occur twice weekly for 6 weeks following the initiation of anti-fungal therapy. The first Week 1 blood sample may be collected within 48 hours of baseline blood collection; however, a minimum of 3 days must occur between all subsequent collections. If patient is released from the hospital prior to the 6-week time point, blood specimens must be collected twice weekly on an out-patient basis up to and including completion of Week 6.

‡‡ Biomarkers specified in Section 2.5.2. Collections must occur once weekly beginning Week 7 and through Week 12. A minimum of 4 days must occur between collections. If patient is released from the hospital prior to the 12-week time point, blood specimens must be collected once weekly on an out-patient basis up to and including completion of Week 12.

§§ Blood collections in addition to the biomarker assays must occur for serum archive and blood profiling assays once at each of the following time points: baseline, Week 2, Week 6, and Week 12.

‖‖ Concomitant medications (beginning 30 days prior to screening) and adverse experiences will be recorded on an ongoing basis throughout the patient's hospitalization. In the event a patient is released from the hospital and continues to be seen on an out-patient basis, concomitant medications and adverse experiences will be recorded weekly prior to blood collections. See Section 3.4 for details of Safety Measurements to be collected in this study.

¶¶ Assessment of Clinical Response must occur at 6 weeks ±7 days and 12 weeks ±7 days from initiation of anti-fungal therapy.

Baseline anatomical imaging study (e.g., CT or MRI) performed within 72 hours prior to, or 24 hours following initiation of anti-fungal therapy is a pre-requisite for enrollment into study. Copies of the baseline anatomical imaging study and report will be provided to the Sponsor.

††† Follow-up anatomical imaging studies (e.g., CT or MRI) will be performed and supported by the Sponsor if an otherwise clinically indicated study has not already or will not be performed ±3 days from the Week 6 or Week 12 clinical outcome assessments. Please see Imaging Operations Manual. Copies of follow-up anatomical imaging study and report will be provided to the Sponsor.

‡‡‡ Study investigator must contact the patient within 24 hours following the last study procedure regardless of when patient discontinues from the study. If a patient has been released from or is unable to visit the hospital, a phone call must be placed.

§§§ Blood collections must occur twice per week during Weeks 1 & 2, and a minimum of 3 days must occur between collections. Blood must also be collected once per week during Week 4. The first Week 1 blood sample may be collected within 48 hours of baseline blood collection.

‖‖‖ Blood collections must occur once per week during Week 6 and Week 12 at the same time that blood is collected for Beta-Glucan and Galactomannan assays.

¶¶¶ Female patients of child-bearing potential must have a negative serum or urine pregnancy test at pre-enrollment (screening) and within 72 hours prior to initiation of anti-fungal therapy.

The patient's fungal infection will be evaluated 14 days after the initiation of systemic anti-fungal therapy. Patients who do not have an established diagnosis of probable or proven IA at 14 days will be discontinued from the study. The results of the assessment must be documented on both the case report form and the Approval for Study Continuation Form.

†††† Physical exams are to be performed by a physician or by qualified personnel under the supervision of a physician. Day 0, Week 6 and Week 12 physical exams must be performed on the same day as the blood draw for biomarker evaluation (beta-glucan, galactomannan, fungal RNA, and fungal DNA).

2. CORE PROTOCOL

2.1 OBJECTIVES AND HYPOTHESES

2.1.1 Primary

Objective

To evaluate whether the average of the z-scores of the time weighted averages of the serial serum measurements of multiple fungal biomarkers during the initial two weeks of anti-fungal therapy can distinguish between successful clinical outcome and failed clinical outcome at 6 weeks in patients with proven or probable invasive aspergillosis.

Hypothesis

In patients with proven or probable invasive aspergillosis, the average of the z-scores of the time weighted averages of the serial serum measurements of (1,3)-β-D-glucan and galactomannan during the initial two weeks of anti-fungal therapy will be lower in patients with a successful clinical outcome compared to patients with a failed clinical outcome. Successful clinical outcome is defined as complete (or partial) cure at 6 weeks from the initiation of anti-fungal therapy.

2.1.2 Secondary

Objectives

1. To evaluate whether the average of the z-scores of the slopes of least-squares straight lines fitted to the serial serum measurements of multiple fungal biomarkers during the initial 2 weeks of anti-fungal therapy can distinguish between successful clinical outcome and failed clinical outcome at 6 weeks in patients with proven or probable invasive aspergillosis.

   Hypothesis

   In patients with proven or probable invasive aspergillosis, the average of the z-scores of the slopes of least-squares straight lines fitted to the serial serum measurements of (1,3)-β-D-glucan and galactomannan during the initial 2 weeks of anti-fungal therapy will be lower (more negative is expected) in patients with a successful clinical outcome compared to patients with a failed clinical outcome. Successful clinical outcome is defined as complete (or partial) cure at 6 weeks from the initiation of anti-fungal therapy.

2. To compare the precision (i.e., effect size) of several composite endpoints of the multiple fungal biomarkers during the initial 2 weeks of anti-fungal therapy (i.e., average z-scores of the time weighted averages, average z-scores of the slopes of least-squares straight lines, average z-scores of the time weighted average changes from baseline, and average z-scores of the percent changes from baseline) with

regards to discriminating between successful clinical outcome and failed clinical outcome at 6 and 12 weeks in patients with proven or probable invasive aspergillosis.

3. To compare the precision (i.e., effect size) of 2 different composite endpoints of the multiple fungal biomarkers during the first week of anti-fungal therapy (i.e., average z-scores of the time weighted averages, and average z-scores of the slopes of least-squares straight lines) with regards to discriminating between successful clinical outcome and failed clinical outcome at 6 and 12 weeks in patients with proven or probable invasive aspergillosis.

4. To compare the precision (i.e., effect size) of 2 different composite endpoints of the multiple fungal biomarkers during the entire course of anti-fungal therapy (i.e., average z-scores of the time weighted averages, and average z-scores of the slopes of least-squares straight lines) with regards to discriminating between successful clinical outcome and failed clinical outcome at 6 and 12 weeks in patients with proven or probable invasive aspergillosis.

2.1.3 Exploratory Objectives

1. To evaluate whether the average of the z-scores of the time weighted averages of the serial serum measurements of multiple fungal biomarkers [(1,3)-β-D-glucan, galactomannan, and fungal RNA] during the initial two weeks of anti-fungal therapy can distinguish between successful clinical outcome and failed clinical outcome at 6 weeks in patients with proven or probable invasive aspergillosis.

2. To evaluate whether the average of the z-scores of the slopes of least-squares straight lines fitted to the serial serum measurements of multiple fungal biomarkers [(1,3)-β-D-glucan, galactomannan, and fungal RNA] during the initial 2 weeks of anti-fungal therapy can distinguish between successful clinical outcome and failed clinical outcome at 6 weeks in patients with proven or probable invasive aspergillosis.

3. To examine the relationship between clinical outcome at 6 and 12 weeks and serial serum measurements of the individual fungal biomarkers in patients with proven or probable invasive aspergillosis.

4. To examine the relationship between serial serum measurements of pairs of fungal biomarkers and clinical outcome at 6 and 12 weeks in patients with proven or probable invasive aspergillosis.

5. To explore various cut points for the fungal biomarker measurements at 2 weeks after initiation of anti-fungal therapy and to explore their sensitivity and specificity for predicting clinical outcome at 6 and 12 weeks in patients with proven or probable invasive aspergillosis.

6. To examine the relationship between patient outcomes based on galactomannan index (GMI) and objective clinical outcome (i.e., survival vs. death).

7. To evaluate additional potential biomarkers that are associated with antifungal treatment response for IA

2.2 PATIENT INCLUSION CRITERIA

1. Patient is a male or female ≥16 years of age at the time informed consent is given.

2. If female, patient is either post-menopausal, free from menses for >2 years, surgically sterilized or willing to use 2 adequate methods of contraception to prevent pregnancy or agrees to abstain from heterosexual activity throughout the study, starting with the pre-enrollment visit.

3. If female patient of childbearing potential, patient must have a negative serum or urine pregnancy test (β-hCG) at pre-enrollment (screening) and within 72 hours of the initiation of anti-fungal therapy.

4. If male, patient is surgically sterilized, agrees to use an adequate method of contraception, or agrees to abstain from heterosexual activity for the duration of the study.

5. Patient is presumed by study investigator to meet definitions of possible, probable or proven invasive aspergillosis as specified in the revised 2008 criteria developed by the European Organization for Research and Treatment of Cancer/Invasive Fungal Infections Co-Operative Group (EORTC/IFICG) and the National Institute of Allergy and Infectious Diseases - Mycoses Study Group (NIAID-MSG): [1] (see Appendix 6.1).

   Proven Invasive Fungal Infection

   Microscopic Analysis: Histopathologic, cytopathologic or direct microscopic examination of a specimen obtained by needle aspiration or biopsy in which hyphae are seen accompanied by evidence of associated tissue damage.

   Culture: Recovery of Aspergillus spp. by culture of a specimen obtained by a sterile procedure from a normally sterile and clinically or radiologically abnormal site consistent with infection, excluding bronchoalveolar lavage fluid, a cranial sinus cavity specimen, and urine.

   Probable Invasive Fungal Infection

   At least 1 host factor criterion (see Appendix 6.1); and 1 clinical criterion; and 1 mycological criterion from abnormal site consistent with infection.

   Possible Invasive Fungal Infection

   At least 1 host factor criterion (see Appendix 6.1); and 1 clinical criterion.

6. Patient must have baseline anatomical imaging study (e.g., CT or MRI) within 72 hours prior to initiation of anti-fungal therapy.

7. Patient understands the study procedures and agrees to participate in the study by giving written informed consent.

8. Patient is willing to comply with the study visit schedule (see Study Flow Chart Section 1.7 for a complete outline of visit requirements).

NOTE: Patients who are participating in another investigational study in which they are receiving anti-fungal therapy with anti-Aspergillus activity are permitted to participate in the study provided they otherwise meet all other eligibility criteria.

2.3 PATIENT EXCLUSION CRITERIA

1. Patient is mentally or legally incapacitated, has significant emotional problems at the time of pre-enrollment (screening) visit or expected during the conduct of the study or has a history of a clinically significant psychiatric disorder over the last 5 to 10 years. Patients who have had situational depression may be enrolled in the study at the discretion of the investigator.

2. Patient has a history of any illness that, in the opinion of the study investigator, might confound the results of the study or poses an additional risk to the patient by their participation in the study.

3. Patient has received hemodialysis using cellulose membranes and/or filters within 2 weeks prior to the pre-enrollment (screening) visit or will receive hemodialysis using cellulose membranes and/or filters during the course of the study.

4. There is any concern by the investigator regarding the safe participation of the patient in the study or for any other reason, the investigator considers the patient inappropriate for participation in the study.

2.4 STUDY DESIGN AND DURATION

2.4.1 Summary of Study Design

This is a prospective, multicenter, non-intervention, observational study enrolling approximately 100 male and female patients ≥16 years of age. In order to be considered for enrollment in the study, patients must:

1) have been identified as a potentially eligible patient by the study investigator; and,

2) have met all eligibility criteria outlined in Sections 2.2 and 2.3; and,

3) have a baseline blood sample collected for biomarker analysis within 24 hours prior to initiation of anti-fungal therapy; and,

4) have had a baseline anatomical imaging study (e.g., CT or MRI) within 72 hours prior to, or 24 hours following initiation of anti-fungal therapy.

Within 14 days after initiation of systemic anti-fungal therapy, enrolled patients must have established a diagnosis of either probable or proven invasive aspergillus infection as defined by the revised 2008 EORTC/MSG criteria in order to be continued in the study. Because the primary objective of this study will evaluate the serial serum measurements of patients with proven or probable IA, patients with an established diagnosis of possible IA after 14 days of initiation of systemic anti-fungal therapy will be discontinued from the study.

Blood will be collected for biomarker analysis at regular intervals while patients are undergoing anti-fungal treatment and enrolled in the study. Pertinent clinical data, including mycological and radiographic information will also be collected during this period. The minimum time period that a patient will be enrolled in the study will be approximately 12 weeks following the initiation of anti-fungal therapy. The data collection period may end earlier in the event of patient death. In the case of patient death, pertinent autopsy information, including cause of death, will also be collected if an autopsy is performed. Patients will be assessed for clinical response at 6 and 12 weeks after initiation of anti-fungal therapy.

2.5 LIST OF MEASUREMENTS

2.5.1 Definitions of Outcome & Clinical Response

Patient outcome will be assessed according to the following definitions for Clinical Response adapted from Segal, et al. [2] Because assessing radiographic improvement, stabilization or worsening of IA is critical to categorizing outcome, the anatomical imaging method used to evaluate outcome will be determined by the study investigator and must be consistent with method used for diagnosis and/or study entry.

2.5.1.1 Successful Outcome: Complete Response (CR)

CR is defined as survival and resolution of all attributable symptoms and signs of infection plus resolution of radiological lesion(s) and documented clearance of infected sites that are accessible to repeat sampling.

If patient death can unambiguously be attributed to causes other than fungal infection (e.g. progressive cancer) **and** above criteria are otherwise fulfilled, cases may be classified as CR.

2.5.1.2 Successful Outcome: Partial Response (PR)

PR is defined as survival and improvement of attributable symptoms and signs of infection plus improvement in attributable radiologic abnormalities and documented clearance of infected sites that are accessible to repeat sampling;

In cases of radiological stabilization (defined as no evidence of progression in attributable radiologic abnormalities), where all attributable symptoms and signs of fungal disease have resolved; or where biopsy of an infected site shows no evidence of hyphae; or where culture is negative, response will be defined as PR.

If patient death can unambiguously be attributed to causes other than fungal infection (e.g. progressive cancer) **and** above criteria are otherwise fulfilled, cases may be classified as PR.

2.5.1.3 Outcome: Failure

Failure is defined as survival and stable response defined as minor or no improvement in attributable symptoms and signs of infection plus radiologic stabilization (defined as no evidence of progression in attributable radiological abnormalities); or persistent isolation of Aspergillus spp or presence of hyphae histologically from infected sites; or evidence of progression of infection defined as worsening clinical symptoms or signs of infection plus new sites of infection or worsening of pre-existing lesions radiologically or persistent isolation of Aspergillus spp. from infected sites; or death.

If patient death can unambiguously be attributed to causes other than fungal infection (e.g. progressive cancer), cases may be classified as CR or PR as stated above.

2.5.2 Biomarkers

Blood will be collected at specific times during the course of the study as detailed in the Study Flow Chart and will be analyzed for the biomarkers listed below which are indicative of invasive Aspergillus infection. The following assays will be used to measure fungal RNA, (1,3)-β-D-glucan, and galactomannan; respectively:

1. NASBA (Nucleic Acid Sequence-Based Amplification) - detects RNA from Aspergillus spp using specific primers selected from 18s rRNA sequences.

2. Beta Glucan Assay (Fungitell®, Associates of Cape Cod, Cape Cod, Massachusetts, USA) - detects (1,3)-β-D-glucan, a cell wall component of many fungi, including Aspergillus spp.

3. Galactomannan Assay (Platelia® Aspergillus, Bio-Rad Laboratories, Hercules, California, USA) - detects the polysaccharide cell-wall component released by *Aspergillus* species during hyphal growth.

4. Profiling assays - quantitative measurement of RNA transcripts in peripheral blood (RNA profiling assays) will be performed at selected time points to explore host factor gene expression changes that may predict or correlate clinical outcome in the setting of invasive Aspergillosis.

In addition, serum will be archived from selected time points. If novel circulating biomarkers of fungal infection are identified in the future, then these biomarkers may be

assayed in these archived serum samples in order to validate the relationship between novel biomarkers and clinical outcome.

2.6 LIST OF SAFETY MEASUREMENTS

Adverse experiences associated with the safety of the procedures specified in Study Flow Chart, Section 1.7 will be assessed through clinical evaluation by the study investigator as part of the patient's standard of care.

2.7 DATA ANALYSIS SUMMARY

Statistical Methods

The primary endpoint of the study is a composite of measurements on multiple biomarkers [(1,3)-β-D-glucan and galactomannan] during the first 2 weeks of anti-fungal therapy with the aim of improving precision for discrimination of clinical success from clinical failure at 6 weeks over that of any of the individual biomarkers. Precision will be measured by effect size (the difference in the means of the average of the z-scores between the group of patients who experienced a clinical success (i.e., complete or partial cure) and the group of patients who experienced a clinical failure, divided by the pooled SD). Prior to the analysis for composites, for each patient, a time weighted average will be calculated using the values obtained within the first 2 weeks (14 days) of the study for each biomarker. O'Brien's global statistic will be computed as the average of the z-scores of the time weighted averages across the biomarkers (where the z-score for each patient's time weighted average for a given biomarker is the difference of each individual patient's time weighted average from the mean, divided by the overall SD of the time weighted averages for the given biomarker). A t-test will be performed to compare the means of the average of the z-scores of the time weighted averages across the biomarkers between patients with a successful clinical outcome at 6 weeks and those with a failed clinical outcome at 6 weeks. A 90% confidence interval for the following true difference: mean of average z-scores of the time weighted averages across the biomarkers for patients with a failed clinical outcome – mean of average z-scores of the time weighted averages across the biomarkers for patients with a successful clinical outcome, will be provided. If the lower bound of the confidence interval is greater than 0 then it can be concluded that the average z-scores of the time weighted averages across the biomarkers are lower in patients with a successful clinical outcome than in patients with a failed clinical outcome.

The endpoint that will be used to test the secondary hypothesis of this study is a composite of multiple biomarkers similar to the primary endpoint. The composite endpoint will be obtained as follows: for each patient, a straight line will be fitted to the values obtained within the first 2 weeks of the study for each biomarker, and a slope will be calculated. O'Brien's global statistic will be computed as the average of the z-scores of the slopes across the biomarkers (where the z-score for each patient's slope for a given biomarker is the difference of each individual patient's slope from the mean, divided by the overall SD of the slopes for the given biomarker). A t-test will be performed to compare the means of the average of the z-scores of the slopes across the biomarkers

between patients with a successful clinical outcome at 6 weeks and those with a failed clinical outcome at 6 weeks. A 90% confidence interval for the following true difference: mean of average z-scores of the slopes across the biomarkers for patients with a failed clinical outcome – mean of average z-scores of the slopes across the biomarkers for patients with a successful clinical outcome, will be provided. If the lower bound of the confidence interval is greater than 0 then it can be concluded that the average z-scores of the slopes across the biomarkers are lower in patients with a successful clinical outcome than in patients with a failed clinical outcome.

Sample Size and Power

The sample size for this study was calculated using information obtained from a retrospective analysis of a small subset of fungal biomarker data from CANCIDAS Protocols (MK0991 Protocols 019 and 037). Protocol 019 was an open, non-comparative trial in which CANCIDAS therapy was given to patients with proven/probable invasive Aspergillus who were refractory to or intolerant of standard anti-Aspergillus therapy. Protocol 037 was an open, non-comparative trial in which CANCIDAS therapy in combination with either a triazole or polyene was given to patients with proven/probable invasive Aspergillus who were refractory to or intolerant of standard anti-Aspergillus therapy. Although these studies were conducted ~10 years ago, serum samples from patients in these studies had been banked and a subset were analyzed for the biomarkers (1,3)-β-D-glucan and galactomannan. Biomarker values and information on clinical outcome were available for 11 patients in Protocol 019 and 11 patients in Protocol 037. Within the first 2 weeks, 21 of the 22 patients had biomarker values for at least 4 time points while 1 patient had biomarker values for 2 time points. Using the methodology described in the "Statistical Methods" paragraph above for the primary hypothesis, an analysis of the composite of the 2 biomarkers was performed and the effect size was computed as the difference in the means of the average of the z-scores of the time weighted averages across the biomarkers between the group of patients who experienced a clinical success (i.e., complete or partial cure) and the group of patients who experienced a clinical failure, divided by the pooled SD. The effect size was computed to be ~0.7. Similarly, using the methodology described in the "Statistical Methods" paragraph above for the secondary hypothesis, the effect size based on the difference in the means of the average of the z-scores of the slopes across the biomarkers between the group of patients who experienced a clinical success and those who experienced a clinical failure was computed to be ~0.5.

In this study, since patients are not being randomized to 2 groups in equal proportions, it is possible that there may be an imbalance between the proportion of patients who experience a clinical success and clinical failure in this study. In the Sims study, 88% of patients experienced a clinical success and the remaining 12% experienced a clinical failure [3]. In the previously mentioned retrospective analysis of a small subset of patients from MK0991 Protocols 019 and 037, 41% of patients experienced a clinical success and the remaining 59% experienced a clinical failure. Assuming that 40% of the patients will experience a clinical success (i.e., complete or partial recovery) and the remaining 60% will experience a clinical failure, with a total sample size of 50 patients

and an assumed true effect size of 0.7 for the primary endpoint described above (i.e., average of the z-scores of the time weighted averages across the biomarkers), there is 78% power for the composite of the 2 biomarkers to discriminate between clinical success and clinical failure at the $\alpha = 0.05$ 1-sided level. Assuming that 50% of the patients will experience a clinical success and the remaining 50% of the patients will experience a clinical failure, with a total sample size of 50 patients and an assumed true effect size of 0.7, there is 80% power for the composite of the 2 biomarkers to discriminate between clinical success and clinical failure at the $\alpha = 0.05$ 1-sided level.

For the secondary hypothesis, assuming that 40% of the patients will experience a clinical success (i.e., complete or partial recovery) and the remaining 60% will experience a clinical failure, with a total sample size of 50 patients and an assumed true effect size of 0.5 for the secondary endpoint of the average of the z-scores of the slopes across the biomarkers, there is 54% power for the composite of the 2 biomarkers to discriminate between clinical success and clinical failure at the $\alpha = 0.05$ 1-sided level.

3. PROTOCOL DETAILS

3.1 RATIONALE

Redacted

Redacted

Redacted

Redacted

Redacted

Redacted

3.2 STUDY PROCEDURES

3.2.1 Informed Consent

3.2.1.1 General Informed Consent

The investigator must obtain documented consent from each potential patient in biomedical research prior to any study related procedures being performed.

Consent must be documented by the patient's dated signature on a Consent Form along with the dated signature of the person conducting the consent discussion. A copy of the signed and dated consent form should be given to the patient before participating in the trial.

For patients that are minors (according to the applicable law that determines the age of majority), a parent or legal guardian must sign the informed consent and the minor patient signs the assent form. The study investigator will determine their competency and capability to be in the study.

3.2.2 Study Restrictions

This study is observational and uses standard-of-care anti-fungal therapy; therefore, study restrictions as applicable to diet, activity, and concomitant medications may vary per patient and are determined by the study investigator.

3.2.2.1 Pregnancy & Contraception

Female patients enrolled in this study should either be post menopausal, free from menses for >2 years, surgically sterilized or willing to use 2 adequate methods of contraception to prevent pregnancy or agree to abstain from heterosexual activity throughout the study, starting with pre-enrollment (screening). Female patients of childbearing potential must have a negative serum or urine pregnancy test (β-hCG) at screening and within 72 hours of initiation of anti-fungal therapy. Male patients enrolled in this study, should also agree to use an adequate method of contraception, be surgically sterilized, or agree to abstain from heterosexual activity for the duration of the study. Patients should be advised to discuss adequate birth control options with the investigator or treating physician.

Patients must be completely informed of the unknown risks of pregnancy and agree not to become pregnant during the time they are participating in this study.

Female patients of childbearing potential enrolled should undergo routine screening for pregnancy prior to CT scans per institutional standard-of-care.

3.2.3 Treatment

This study is observational and uses standard-of-care anti-fungal therapy; therefore, the investigator shall use discretion in determining the appropriate timing for initiation and duration of therapy for an individual patient.

3.2.4 Procedures

Study procedures should be completed as close to the prescribed/scheduled time as possible. Procedures will be performed in the following order of proximity (below) with regard to the prescribed time. These procedures can be done prior to or after the timepoint.

1. Blood collection for biomarker assays, serum archives, and profiling assays (see Appendix 6.2 for blood volumes)
2. Vital signs (Oral temperature, BP, HR, RR. Weight will also be measured at all times vital signs are assessed)
3. Physical examinations

The order of priority can be changed during the study with joint agreement of the investigator and the Sponsor Clinical Monitor.

Any nonscheduled procedures required for urgent evaluation of safety concerns take precedence over all routine scheduled procedures.

For all blood sample collections, usage of cotton-containing gauze, swabs, pads and bandages must be avoided. In addition, powder-free gloves must be worn by the phlebotomist. Powder-containing gloves must not be used to collect blood for biomarker assays.

3.2.4.1 Pre-Enrollment Period

All potential patients will be identified by the study investigator and are anticipated to be already hospitalized during the screening/enrollment period.

Procedures to be performed within 72 hours following initial identification of the potential patient by the study investigator:

1. The investigator shall discuss, and provide in writing, the nature of the study and its requirements/restrictions so as to obtain written informed consent prior to participation in the study.

2. The study investigator must obtain a complete medical history, including a detailed description of the history of the infection, and date of onset of signs and symptoms of infection at initial presentation. The date of the onset of symptoms and the nature of the patient's underlying disease must also be documented in the source and on the case report form. Documentation of initial diagnosis of IA including radiographs, microscopic analysis, culture results, non-culture based results and invasive procedures should be provided if available as relevant to eligibility criteria. (Results of baseline anatomical imaging study, e.g., CT or MRI, must be provided.)

3. The study investigator must record all prior and concomitant medications taken 30 days prior to screening on the case report form. All antibiotic and/or antifungal medications taken prior to the initiation of systemic anti-fungal therapy, including all prior anti-fungal therapy for IA, and whether or not the patient was considered refractory or intolerant of prior therapy must be documented in the source and on the case report form.

4. Patients will undergo a complete physical examination performed by a physician or by qualified personnel under the supervision of a physician, including a detailed description of the nature and extent of infection. The status of the patient’s underlying disease, as well as identification of any predisposing risk factors for IA, must be documented in the source and on the case report form.

5. Potential patients will be evaluated to determine whether they fulfill the entry requirements set forth in Sections 2.2 and 2.3.

See Section 1.7 Study Flow Chart for additional details on Pre-Enrollment Period procedures.

3.2.4.2 Day 0

Timing of initiation of anti-fungal therapy is to be determined by the study investigator. The Study Period will begin when the patient begins systemic anti-fungal therapy. A baseline blood sample for biomarker analysis, serum archives, and profiling assays should be collected within 24 hours prior to the initiation of anti-fungal therapy. If a pre-therapy sample cannot be obtained, baseline blood sample must be collected within 24 hours of initiation of anti-fungal therapy. Upon collection of a baseline blood sample for biomarker analysis, patients will be considered enrolled in the study. Details regarding blood collection, processing, storage and shipment procedures will be provided in a separate manual. See Appendix 6.2 for blood volume collection requirements.

In the event of patient death, pertinent autopsy information, including cause of death, will be collected if an autopsy is performed.

See Section 1.7 Study Flow Chart for additional details on Day 0 procedures.

3.2.4.3 Weeks 1-5

Patients will be assessed for potential adverse experience(s) and any change(s) in relevant medical history and concomitant medications.

All results including radiographs, microscopic analysis, culture results, non-culture based results and invasive procedures should be provided if relevant to patient's aspergillosis infection.

Vital signs and signs and symptoms attributable to IA will be assessed. Blood will be collected for (1,3)-β-D-glucan and galactomannan biomarker assays, profiling assays, serum archives, and analysis of fungal RNA and fungal DNA according to the schedule outlined in Section 1.7. Please note, if necessary, the first set of blood samples to be drawn during Week 1 may be collected within 48 hours of baseline blood collection; however, a minimum of 3 days must occur between all subsequent collections.

The patient’s fungal infection will be evaluated 14 days after the initiation of systemic anti-fungal therapy. Patients with an established diagnosis of possible IA at 14 days will be discontinued from the study. The results of the assessment must be documented on both the case report form and the Approval for Study Continuation (ASC) Form. For patients with an established diagnosis of probable or proven IA at 14 days, sites are expected to provide appropriate supporting documentation.

In the event of patient death, pertinent autopsy information, including cause of death, will be collected if an autopsy is performed.

See Section 1.7 Study Flow Chart for additional details on procedures to occur during Weeks 1-5.

3.2.4.4 Week 6

At 6 weeks ±7 days after initiation of anti-fungal therapy, patients will be evaluated. Depending on patient's clinical status, the patient may be receiving or may have completed anti-fungal therapy.

Patients will be assessed for potential adverse experience(s) and any change(s) in relevant medical history and concomitant medications.

A complete physical examination and overall clinical assessment, including vital signs, and a detailed description/evaluation of the attributable signs/symptoms of infection and underlying disease will be performed.

Blood will be collected for (1,3)-β-D-glucan and galactomannan assays twice during Week 6 with a minimum of 3 days between collections. In addition, a follow-up anatomical imaging study (e.g., CT or MRI) to assess radiographic response will be performed and supported by the Sponsor if a clinically indicated study has not already nor will be performed ±7 days of the Week 6 assessment. Other evaluations will be conducted at the discretion of the study investigator as per standard-of-care.

Blood must also be collected once during Week 6 for serum archives, profiling assays, and analysis of fungal RNA and fungal DNA.

Response should be documented by assessment of attributable symptoms, radiographic studies, and by culture and/or microscopic analysis if applicable. Because radiographic improvement/progression of IA is critical to assessing response to therapy, radiographic studies positive at diagnosis and/or study entry must be repeated at the Week 6 assessment as stated above.

In the event of patient death, pertinent autopsy information, including cause of death, will be collected if an autopsy is performed.

See Section 1.7 Study Flow Chart for additional details on Week 6 procedures.

3.2.4.5 Weeks 7-11

Depending on patient's clinical status, the patient may be receiving or may have completed anti-fungal therapy.

Patients will be assessed for potential adverse experience(s) and any change(s) in relevant medical history and concomitant medications.

All results including radiographs, microscopic analysis, culture results, non-culture based results and invasive procedures should be provided if relevant to patient's aspergillosis infection.

Vital signs and signs and symptoms attributable to IA will be assessed and blood will be collected for biomarker assays once weekly with a minimum of 4 days between assessment/collection time points.

In the event of patient death, pertinent autopsy information, including cause of death, will be collected if an autopsy is performed.

See Section 1.7 Study Flow Chart for additional details on procedures to occur during Weeks 7-11.

3.2.4.6 Week 12

At 12 weeks after initiation of anti-fungal therapy, patients will be evaluated. Depending on patient's clinical status, the patient may be receiving or may have completed anti-fungal therapy.

Patients will be assessed for potential adverse experience(s) and any change(s) in relevant medical history and concomitant medications.

A complete physical examination and overall clinical assessment, including vital signs, detailed description and evaluation of the attributable signs/symptoms of infection and underlying disease will be performed.

Blood samples will be collected once for biomarker assays (serum archives, and profiling assays during Week 12), and a follow-up anatomical imaging study (e.g., CT or MRI) to assess radiographic response will be performed and supported by the Sponsor if a clinically indicated study has not already nor will be performed ±7 days of the Week 12 assessment. Other evaluations will be conducted at the discretion of the study investigator as per standard-of-care.

Response should be documented by assessment of attributable symptoms, radiographic studies, and by culture and/or microscopic analysis if applicable. Because radiographic improvement/progression of IA is critical to assessing response to therapy, radiographic studies positive at diagnosis and/or study entry must be repeated at the Week 12 assessment as stated above.

In the event of patient death, pertinent autopsy information, including cause of death, will be collected if an autopsy is performed.

See Section 1.7 Study Flow Chart for additional details on Week 12 procedures.

3.2.4.7 Follow-up

The study investigator must contact the patient 24 hours following the last study procedure to assess for potential adverse experience(s) and any change(s) in relevant medical history and concomitant medications. Follow-up must occur regardless of when patient is discontinued from the study, except in the event of patient death.

In the event of patient death, pertinent autopsy information, including cause of death, will be collected if an autopsy is performed.

3.2.4.8 Assignment of Baseline Number/Screening

Patients will be assigned a baseline number at the screening visit.

3.2.4.9 Allocation

Patients will not be randomized in this study. If the patient meets all screening criteria the assigned baseline number will become the patient's allocation number. The allocation number will be used to identify the patient for all procedures occurring after enrollment.

A single patient/subject cannot be assigned more than 1 allocation number.

3.2.4.10 Discontinuation/Withdrawal from Study

Because the primary objective of this study will evaluate the serial serum measurements of patients with proven or probable IA, patients who do not have an established diagnosis of probable or proven IA after 14 days of baseline blood collection will be discontinued from the study.

Subjects/patients may withdraw at any time or be dropped from the study at the discretion of the investigator should any untoward effects occur. In addition, a subject/patient may be withdrawn by the investigator or the SPONSOR if he/she violates the study plan or for administrative and/or other safety reasons. The investigator or study coordinator must notify the SPONSOR immediately when a subject/patient has been discontinued/ withdrawn due to an adverse experience (telephone or FAX). When a subject/patient discontinues/withdraws prior to study completion, all applicable activities scheduled for the final study visit should be performed at the time of discontinuation. Any adverse experiences which are present at the time of discontinuation/withdrawal should be followed in accordance with the safety requirements outlined in Section 3.4 SAFETY MEASUREMENTS - DETAILS.

Subjects/patients who discontinue from the study for reasons unrelated to the study (e.g., personal reasons) will be replaced as required for the study to meet its objectives. The decision to remove a subject/patient and to replace dropouts will be made jointly by the investigator, SPONSOR Clinical Monitor, and SPONSOR study statistician. Both the replacement and originally allocated number will be unique numbers.

3.3 EFFICACY MEASUREMENTS

3.3.1 Clinical and Laboratory Measurements for Efficacy

3.3.1.1 Fungitell™ Assay

The Fungitell™ assay measures levels of (1,3)-β-D-glucan in serum. The detection reagent is a biological cascade based upon modified Limulus Amebocyte Lysate (LAL), an extract of the blood cells of the North American Horseshoe Crab. The assay is based upon a modification of the LAL pathway. The Fungitell reagent is modified to eliminate

Factor C and, thus bypassing the activated factor B, to only react to (1,3)-β-D-glucan, through the Factor G-mediated side of the pathway (See Figure 3-4). This renders the reagent highly specific for (1,3)-β-D-glucan and does not react to other polysaccharides, including beta-glucans with different glycosidic linkages.

Figure 3-4

Enzyme Cascade

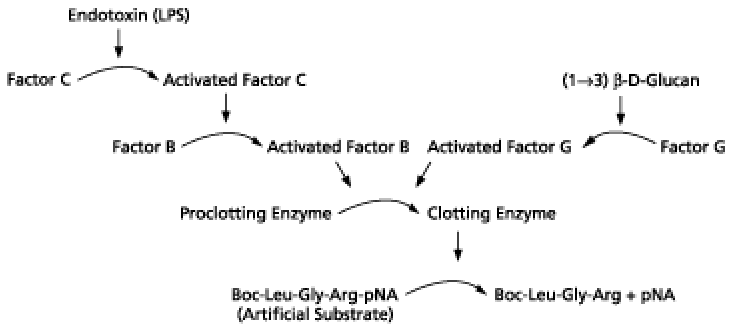

When (1,3)-β-D-glucan is present in a sample, it activates Factor G, a serine protease zymogen. The activated Factor G converts the inactive proclotting enzyme to the active clotting enzyme, which in turn cleaves pNA from the chromogenic peptide substrate, Boc-Leu-Gly-Arg-pNA, creating a chromophore that absorbs at 405 nm. The reagent is used in the Fungitell kinetic assay, to detect the rate of optical density increase in a sample. This rate is interpreted against a standard curve to produce estimates of (1,3)-β-D-glucan concentration in the sample.

Similar to enzyme immunoassays, the Fungitell assay is performed in microplates and read in an incubating reader. It is a homogeneous assay, and does not require any washing steps. It can be completed in approximately 1 hour.

3.3.1.2 Galactomannan Assay

The Bio-Rad Laboratories Platelia® *Aspergillus* EIA is a one-stage immunoenzymatic sandwich microplate assay that uses rat monoclonal antibody EBA-2 to detect circulating galactomannan antigen (GM). Results can be obtained in three hours.

When used in conjunction with other diagnostic procedures, Platelia *Aspergillus* EIA is an aid in the diagnosis of Invasive Aspergillosis in immunocompromised patients undergoing hematopoietic stem cell transplantation and in patients with hematological malignancy.

3.3.1.3 NASBA Assay

Validation of the pan-*Aspergillus* 28S rRNA NASBA assay was performed using naïve blood spiked with either purified *A. fumigatus* total RNA or *A. fumigatus* spores. The sequence of the primers and the molecular beacon probe, as well as the NASBA reaction

conditions, are as described in Zhao et al [17]. The detection limit for purified *A. fumigatus* total RNA added directly to NASBA reactions was 5 pg, and the assay had a linear range that spanned at least four $\log_{10}$ (Figure 3-5).

Figure 3-5

Sensitivity of the 28S Pan-*Aspergillus* NASBA Assay

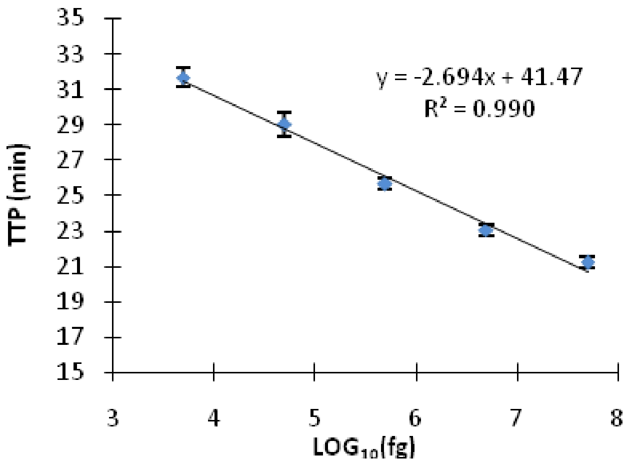

A. fumigatus total RNA was serially diluted and added to NASBA reactions. The time to positivity (TTP) reflects the time required for the fluorescence signal to exceed a minimum threshold established in template-free reactions run in parallel.

The protocol for quantifying *Aspergillus* RNA in whole blood was tested by spiking either *A. fumigatus* total RNA or *A. fumigatus* spores into PAXgene™ Blood RNA tubes containing whole blood. Total RNA was purified from the PAXgene™ nucleic acid pellet using chemical and mechanical lysis, followed by NucliSENS easyMAG extraction and isolation procedures [16]. Detection limits of 100 pg of *A. fumigatus* free total RNA or 100 *A. fumigatus* spores per 2.5 ml of blood were established in these experiments (Figure 3-6 and Figure 3-7).

Figure 3-6

28S Pan-*Aspergillus* NASBA Assay Using Total *A. fumigatus* RNA Spiked in Lysed Blood Pellet

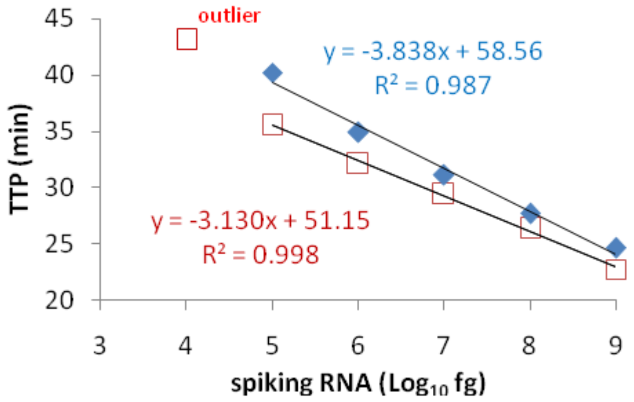

Serial dilutions of *A. fumigatus* total RNA were added to the nucleic acid pellets recovered following centrifugation of 2.5 mL of blood collected in PAXgene™ Blood RNA tubes. Pan-*Aspergillus* NASBA reactions were performed on total nucleic acid recovered from each spiked blood pellet, and the time to positivity (TTP) was determined. The standard nucleic acid isolation method (NucliSENS easyMAG; open red squares) was compared to an alternate method (filled blue diamonds) in this experiment.

Figure 3-7

28S Pan-*Aspergillus* NASBA Assay Using *A. fumigatus* Conidia Spiked in Lysed Blood Pellet

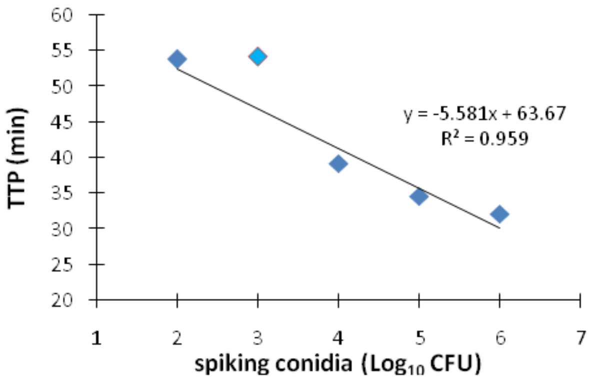

Serial dilutions of *A. fumigatus* spores (conidia) were added to nucleic acid pellets recovered following centrifugation of 2.5 mL of blood collected in PAXgene™ Blood RNA tubes. Total nucleic acid was extracted and isolated using the NucliSENS easyMAG method, and the time to positivity (TTP) in pan-*Aspergillus* NASBA assays was determined.

This pan-*Aspergillus* NASBA assay is suitable for detection of ≥ 100 pg of purified *Aspergillus* total RNA or ≥ 100 *Aspergillus* spores in 2.5 ml of blood collected in a PAXgene™ Blood RNA tube. Both assays have a linear dynamic range up to four $\log_{10}$ above the lower limit of detection; therefore, quantitative signals should be detectable in blood samples from patients whose *Aspergillus* RNA levels fall in the range of 4×10^1 to 4×10^5 pg/mL blood, or whose *Aspergillus* spore levels are in the range of 4×10^1 to 4×10^5 spores/mL blood. Samples with a NASBA TTP below the minimum threshold of the assay (i.e. RNA quantities above the linear range) can be diluted to provide an on-scale value.

3.3.1.4 Anatomical Imaging Procedures

Please refer to Imaging Operations Manual for requirements for conducting anatomical imaging studies (e.g., CT or MRI).

3.3.2 Adjudication Procedures

Six- and twelve-week clinical outcome assessments will be performed by up to three blinded clinicians based solely on the following information: 1) clinical data, including signs and symptoms of infection, 2) mycological data, and 3) radiographic study results. These individuals will not have access to any fungal biomarker measurement data obtained during the study.

Further, radiographic studies will be reviewed by up to three blinded licensed radiologists. Baseline, 6-week, and 12-week images will be presented to reviewers for comparison and determination of resolution, improvement, stabilization, or worsening.

Roles and responsibilities of the Independent Adjudication Board (IAB) and other operational procedures performed by the IAB, including guidelines for interpretation of radiographic images by the IAB, are outlined in a separate Charter.

3.4 SAFETY MEASUREMENTS

3.4.1 Clinical and Laboratory Measurements for Safety

Adverse experiences associated with the safety of the procedures specified in Study Flow Chart, Section 1.7 will be assessed through clinical evaluation by the study investigator as part of the patient's standard of care.

3.4.2 Recording Adverse Experiences

An adverse experience is defined as any unfavorable and unintended change in the structure, function, or chemistry of the body temporally associated with protocol specified procedure, whether or not considered related to the use of the product. Any worsening (i.e., any clinically significant adverse change in frequency and/or intensity) of a preexisting condition which is temporally associated with protocol specified procedure, is also an adverse experience.

Changes resulting from normal growth and development which do not vary significantly in frequency or severity from expected levels are not to be considered adverse experiences. Examples of this may include, but are not limited to, teething, typical crying in infants and children, and onset of menses or menopause occurring at a physiologically appropriate time.

Adverse experiences may occur in the course of the use of a protocol specified procedure in clinical studies or within the follow-up period specified by the protocol, or prescribed in clinical practice, from overdose (whether accidental or intentional), from abuse, and from withdrawal.

Adverse experiences may also occur in screened subjects/patients during any preallocation baseline period as a result of a protocol-specified intervention including washout or discontinuation of usual therapy, diet, placebo treatment, or a procedure.

Such events will be recorded at each examination on the Adverse Experience Case Report Forms/Worksheets.

3.4.3 Reporting of Pregnancy to SPONSOR

Although not considered an adverse experience, it is the responsibility of investigators or their designees to report any pregnancy in a subject/patient (spontaneously reported to them) which occurs during the study or within 14 days of completing the study. All subjects/patients who become pregnant must be followed to the completion/termination of the pregnancy. If the pregnancy continues to term, the outcome (health of infant) must also be reported to one of the individuals listed on the SPONSOR Contact Information page found in the Administrative Binder.

3.4.4 Immediate Reporting of Adverse Experiences to the SPONSOR

3.4.4.1 Serious Adverse Experiences

Any serious adverse experience, including death related to the protocol specified procedure (s) or pharmaceutical products used for the procedure (s) which occurs to any subject/patient entered into this study within 5 days following the procedure, must be reported within 24 hours to one of the individual(s) listed on the contact information page. Additionally, any serious adverse experience considered by an investigator who is a qualified physician to be possibly, probably, or definitely related to the protocol specified procedure that is brought to the attention of the investigator at any time outside of the time period specified in the previous paragraph also must be reported immediately to one of the individuals listed on the sponsor contact information page found in the administrative binder.

All subjects/patients with serious adverse experiences related to the protocol specified procedure(s) or participation in the study must be followed to outcome.

The investigator must report serious adverse experiences related to any pharmaceutical product that is not considered a test drug(s), but is being used while participating in this study, to the manufacturer of that product.

3.4.5 Evaluating Adverse Experiences

Refer to Table 3-1 for instructions in evaluating adverse experiences.

Table 3-1

An investigator who is a qualified physician, will evaluate all adverse experiences as to:

**Maximum Intensity**	**Mild**	awareness of sign or symptom, but easily tolerated (for pediatric studies, awareness of symptom, but easily tolerated)
	**Moderate**	discomfort enough to cause interference with usual activity (for pediatric studies, definitely acting like something is wrong)
	**Severe**	incapacitating with inability to work or do usual activity (for pediatric studies, extremely distressed or unable to do usual activities)
**Seriousness**	A serious adverse experience is any adverse experience occurring at any dose that:	
	†**Results in death**; or	
	†**Is life threatening; or** places the subject/patient, in the view of the investigator, at immediate risk of death from the experience as it occurred [Note: This does not include an adverse experience that, had it occurred in a more severe form, might have caused death.]; or	
	†**Results in a persistent or significant disability/incapacity** (substantial disruption of one's ability to conduct normal life functions); or	
	†**Results in or prolongs an existing inpatient hospitalization** (hospitalization is defined as an inpatient admission, regardless of length of stay, even if the hospitalization is a precautionary measure for continued observation. (Note: Hospitalization [including hospitalization for an elective procedure] for a preexisting condition which has not worsened does not constitute a serious adverse experience.); or	
	†**Is a congenital anomaly/birth defect** (in offspring of subject/patient taking the product regardless of time to diagnosis); or	
	**Is a cancer;** or	
	**Is an overdose** (Whether accidental or intentional.) Any overdose whether or not associated with an adverse experience must be reported within 24 hours to one of the individuals on the Contact Information Page found in the Administrative Binder.	
	**Other important medical events** that may not result in death, not be life threatening, or not require hospitalization may be considered a serious adverse experience when, based upon appropriate medical judgment, the event may jeopardize the subject/patient and may require medical or surgical intervention to prevent one of the outcomes listed previously (designated above by a †).	
**Duration**	Record the start and stop dates of the adverse experience. If less than 1 day, indicate the appropriate length of time and units	
**Action taken**	Did the adverse experience cause the protocol specified procedure to be discontinued?	
**Relationship to protocol specified procedure**	Did the protocol specified procedure cause the adverse experience? The determination of the likelihood that the protocol specified procedure caused the adverse experience will be provided by an investigator who is a qualified physician. The investigator's signed/dated initials on the source document or worksheet, that supports the causality noted on the AE form, ensures that a medically qualified assessment of causality was done. This initialed document must be retained for the required regulatory time frame. The criteria below are intended as reference guidelines to assist the investigator in assessing the likelihood of a relationship between the protocol specified procedure and the adverse experience based upon the available information.<br>**The following components are to be used to assess the relationship between the protocol specified procedure and the AE**; the greater the correlation with the components and their respective elements (in number and/or intensity), the more likely the protocol specified procedure caused the adverse experience (AE):	
	**Exposure**	Is there evidence that the subject/patient was actually exposed to the protocol specified procedure such as: reliable history, acceptable compliance assessment (pill count, diary, etc.), expected pharmacologic effect, or measurement of drug/metabolite in bodily specimen?
	**Time Course**	Did the AE follow in a reasonable temporal sequence from administration of the protocol specified procedure?<br>Is the time of onset of the AE compatible with a drug-induced effect?
	**Likely Cause**	Is the AE not reasonably explained by another etiology such as underlying disease, other drug(s)/vaccine(s), or other host or environmental factors

**Relationship to protocol specified procedure (continued)**	**The following components are to be used to assess the relationship between the protocol specified procedure and the AE: (continued)**	
	**Dechallenge**	Was the protocol specified procedure discontinued or frequency reduced?<br>If yes, did the AE resolve or improve?<br>If yes, this is a positive dechallenge. If no, this is a negative dechallenge.<br>(Note: This criterion is not applicable if: (1) the AE resulted in death or permanent disability; (2) the AE resolved/improved despite continuation of the protocol specified procedure; or (3) the study is a single-dose drug study.)
	**Rechallenge**	Was the subject/patient reexposed to the protocol specified procedure in this study?<br>If yes, did the AE recur or worsen?<br>If yes, this is a positive rechallenge. If no, this is a negative rechallenge.<br>(Note: This criterion is not applicable if: (1) the initial AE resulted in death or permanent disability, or (2) the study is a single-dose drug study.)<br>NOTE: IF A RECHALLENGE IS PLANNED FOR AN ADVERSE EVENT WHICH WAS SERIOUS AND WHICH MAY HAVE BEEN CAUSED BY THE PROTOCOL SPECIFIED PROCEDURE, OR IF REEXPOSURE TO THE PROTOCOL SPECIFIED PROCEDURE POSES ADDITIONAL POTENTIAL SIGNIFICANT RISK TO THE SUBJECT/PATIENT, THEN THE RECHALLENGE MUST BE APPROVED IN ADVANCE BY THE U.S. CLINICAL MONITOR AND THE INSTITUTIONAL REVIEW BOARD/INDEPENDENT ETHICS COMMITTEE.
	**Consistency with Study Drug Profile**	Is the clinical/pathological presentation of the AE consistent with previous knowledge regarding the protocol specified procedure or drug class pharmacology or toxicology?
	The assessment of relationship will be reported on the case report forms/worksheets by an investigator who is a qualified physician according to his/her best clinical judgment, including consideration of the above elements.<br>**Use the following scale of criteria as guidance (not all criteria must be present to be indicative of a drug relationship).**	
	**Definitely related**	There is evidence of exposure to the protocol specified procedure. The temporal sequence of the AE onset relative to administration of the protocol specified procedure is reasonable. The AE is more likely explained by the protocol specified procedure than by another cause. Dechallenge is positive. Rechallenge (if feasible) is positive. The AE shows a pattern consistent with previous knowledge of the protocol specified procedure or protocol specified procedure class.
	**Probably related**	There is evidence of exposure to the protocol specified procedure. The temporal sequence of the AE onset relative to administration of the protocol specified procedure is reasonable. The AE is more likely explained by the protocol specified procedure than by another cause. Dechallenge (if performed) is positive.
	**Possibly related**	There is evidence of exposure to the protocol specified procedure. The temporal sequence of the AE onset relative to administration of the protocol specified procedure is reasonable. The AE could have been due to another equally likely cause. Dechallenge (if performed) is positive.
	**Probably not related**	There is evidence of exposure to the protocol specified procedure. There is another more likely cause of the AE. Dechallenge (if performed) is negative or ambiguous. Rechallenge (if performed) is negative or ambiguous.
	**Definitely not related**	The subject/patient did not receive the protocol specified procedure. **OR** Temporal sequence of the AE onset relative to administration of the protocol specified procedure is not reasonable. **OR** There is another obvious cause of the AE.

3.4.6 SPONSOR Responsibility for Reporting Adverse Experiences

All adverse experiences will be reported to regulatory agencies, IRB/IECs, and investigators in accordance with all applicable global laws and regulations.

3.5 DATA ANALYSIS

This section outlines the statistical analysis strategy and procedures for the study. If, after the study has begun, changes are made to the statistical analysis plan stated below, then these deviations to the plan will be listed, along with an explanation as to why they occurred, in the final study report for the study, as appropriate. Post hoc exploratory analyses will be clearly identified in the final study report.

3.5.1 Responsibility for Analyses

The statistical analysis of the data obtained from this study will be the responsibility of the Experimental Medicine Statistics department of the SPONSOR.

3.5.2 Hypotheses/Estimation

Objectives and hypotheses of the study are stated in Section 2.1.

3.5.3 Analysis Endpoints

Primary

The primary time point for evaluation of clinical outcome is 6 weeks after initiation of anti-fungal therapy in patients with proven or probable invasive aspergillosis.

The primary endpoint of the study is a composite of the measurements on multiple biomarkers [(1,3)-β-D-glucan and galactomannan] during the initial two weeks (14 days) of anti-fungal therapy. The metric for combining the multiple measurements across the first 2 weeks of anti-fungal therapy for each biomarker is the time weighted average across the first 2 weeks of anti-fungal therapy. The composite endpoint will be formed by calculating the average of the z-scores of the time weighted averages across the biomarkers for each patient (i.e., O'Brien's global statistic). The z-score for each patient's time weighted average for a given biomarker is the difference of each individual patient's time weighted average from the mean, divided by the overall SD of the time weighted averages for the given biomarker.

Secondary

For the secondary and exploratory analyses, clinical outcome will be assessed at 6 weeks and 12 weeks after initiation of anti-fungal therapy.

The endpoint for the secondary hypothesis of this study is a composite of multiple biomarkers [(1,3)-β-D-glucan and galactomannan] similar to the primary endpoint. The composite endpoint will be obtained as follows: for each patient, a straight line will be fitted to the values obtained within the first 2 weeks of the study for each biomarker, and a slope will be calculated. O'Brien's global statistic will be computed as the average of

the z-scores of the slopes across the biomarkers (where the z-score for each patient's slope for a given biomarker is the difference of each individual patient's slope from the mean, divided by the overall SD of the slopes for the given biomarker).

Other secondary endpoints of this study are as follows:

1. Average z-scores of the time weighted average changes from baseline computed as follows: for each patient's post baseline values obtained within the first 2 weeks of the study, the differences (post baseline – baseline) will be computed and the time weighted averages across these differences will be computed for each biomarker. O'Brien's global statistic will be computed as the average of the z-scores of the time weighted average changes from baseline across the biomarkers (where the z-score for each patient's time weighted average for a given biomarker is the difference of each individual patient's time weighted average from the mean, divided by the overall SD of the time weighted averages for the given biomarker).

2. Average z-scores of the percent changes from baseline computed as follows: for each patient, the percent change from baseline at 2 weeks after initiation of anti-fungal therapy will be computed for each biomarker. O'Brien's global statistic will be computed as the average of the z-scores of the percent changes from baseline across the biomarkers (where the z-score for each patient's percent change from baseline for a given biomarker is the difference of each individual patient's percent change from baseline from the mean, divided by the overall SD of the percent changes from baseline for the given biomarker).

The primary endpoint of the average z-scores of the time weighted averages across the biomarkers and the secondary endpoint of the average z-scores of the slopes of least-squares straight lines across the biomarkers will also be computed over the following time periods: 1) the first week of anti-fungal therapy (7 days), and 2) throughout the entire course of anti-fungal therapy.

Exploratory

The exploratory endpoints of the study are as follows:

1. Composite endpoint of the time weighted averages of multiple biomarkers during the initial two weeks of anti-fungal therapy, similar to the primary endpoint, but including the following biomarkers: (1,3)-β-D-glucan, galactomannan, and fungal RNA.
2. Composite endpoint of the slopes of least-squares straight lines fitted to the serial serum measurements of multiple fungal biomarkers during the initial two weeks of anti-fungal therapy, similar to the secondary endpoint, but including the following biomarkers: (1,3)-β-D-glucan, galactomannan, and fungal RNA.
3. For each individual biomarker, time weighted average, slope of straight line fitted to the data, time weighted average changes from baseline, and percent change from baseline computed over the following time periods: 1) the first week of anti-fungal

therapy, 2) the first 2 weeks of anti-fungal therapy, and 3) throughout the entire course of anti-fungal therapy.

4. Composite endpoints of the time weighted averages of pairs of biomarkers similar to the primary endpoint where the time weighted averages are calculated over the following time periods: 1) the first week of anti-fungal therapy, 2) the first 2 weeks of anti-fungal therapy, and 3) throughout the entire course of anti-fungal therapy, in patients with proven or probable invasive aspergillosis.
5. Various cut points for the fungal biomarker measurements at 2 weeks after initiation of anti-fugal therapy determined based on the data.
6. Patient outcomes based on galactomannan index (GMI) will be defined as follows: Patient will be defined as a success if (1) patient is persistently GMI negative (GMI<0.5) for at least 14 days after the first negative value, and (2) patient is alive at the end of the 14 day period, and (3) if patient does not show evidence of aspergillosis on tests or procedures performed (if any) at the time of outcome determination. If any of the above 3 criteria are not satisfied, the patient will be defined as a failure.

3.5.4 Analysis Populations

The primary analysis population will be the per-protocol population. This population will include patients who fulfill the following criteria:

- Patients with a diagnosis of proven or probable invasive aspergillosis
- Patients who survive for at least 2 weeks after initiation of anti-fungal therapy
- Patients with a clinical outcome determined at 6 weeks (except if a patient dies before 6 weeks in which case the patient will be included in the analysis as a clinical failure. However, if the death can unambiguously be attributed to causes other than fungal infection (e.g. progressive cancer) then the patient may be classified as a complete/partial responder if he/she fulfills the criteria in the definition of a complete/partial responder)
- Patients with at least 2 valid results for each biomarker, i.e., one initial value and one subsequent value within the appropriate time period.

3.5.5 Statistical Methods

Primary Analysis

The primary endpoint of the study is a composite of measurements on multiple biomarkers [(1,3)-β-D-glucan and galactomannan] during the first 2 weeks of anti-fungal therapy with the aim of improving precision for discrimination of clinical success from clinical failure at 6 weeks over that of any of the individual biomarkers. Precision will be measured by effect size (the difference in the means of the average of the z-scores between the group of patients who experienced a clinical success (i.e., complete or partial

cure) and the group of patients who experienced a clinical failure, divided by the pooled SD). Prior to the analysis for composites, for each patient, a time weighted average will be calculated using the values obtained within the first 2 weeks (14 days) of the study for each biomarker. O'Brien's global statistic will be computed as the average of the z-scores of the time weighted averages across the biomarkers (where the z-score for each patient's time weighted average for a given biomarker is the difference of each individual patient's time weighted average from the mean, divided by the overall SD of the time weighted averages for the given biomarker). A t-test will be performed to compare the means of the average of the z-scores of the time weighted averages across the biomarkers between patients with a successful clinical outcome at 6 weeks and those with a failed clinical outcome at 6 weeks. A 90% confidence interval for the following true difference: mean of average z-scores of the time weighted averages across the biomarkers for patients with a failed clinical outcome – mean of average z-scores of the time weighted averages across the biomarkers for patients with a successful clinical outcome, will be provided. If the lower bound of the confidence interval is greater than 0 then it can be concluded that the average z-scores of the time weighted averages across the biomarkers are lower in patients with a successful clinical outcome than in patients with a failed clinical outcome.

Analyses to address the secondary hypothesis test and all other secondary and exploratory objectives involving multiple fungal biomarkers will use methodology similar to that described above for the primary analysis.

Sensitivity Analyses

A sensitivity analysis will be performed in which those patients who have a clinical outcome assessed prior to 6 weeks from initiation of anti-fungal therapy will be included. The analysis method will be the same as that described for the primary analysis.

A sensitivity analysis may be considered in which patients with a known history of or known exposure to therapies, or with concurrent conditions that are known to have an impact on the (1,3)-β-D-glucan and galactomannan assays will be excluded from the analysis. Both concurrent and prior therapy with β-lactam antibiotics have been reported to cause false positive results for the galactomannan assay, whereas prior mold-active antifungal treatment for prophylaxis has been shown to reduce the sensitivity of galactomannan testing. False positive results for (1,3)-β-D-glucan testing have been reported in certain bacteremic patients and serum exposure to cellulose membranes and cotton bandage. High concentrations of bilirubin or triglycerides have also been reported as inhibitory in the assay. The analysis method will be the same as that described for the primary analysis.

Exploratory Analyses

To assess the exploratory objective of examining the relationship between clinical outcome and serial serum measurements of the individual fungal biomarkers, the analysis will be performed using the following composite measures of the biomarkers: time

weighted average, slope of straight line fitted to the data, time weighted average changes from baseline, and percent change from baseline, for each of the fungal biomarkers separately. These endpoints will be computed as mentioned in Section 3.5.3. For each biomarker, each of these endpoints will be analyzed for each time period via a t-test to compare the means of the endpoint between patients with a successful clinical outcome and those with a failed clinical outcome.

Correlations between individual biomarker values and clinical outcome may be computed for biomarker values at 1 week and 2 weeks following initiation of anti-fungal therapy.

Logistic regression models may be used to further explore the relationship between the various biomarkers and clinical outcome. Clinical outcome will be the response variable and the values at 2 weeks following initiation of anti-fungal therapy for each biomarker will be the explanatory variables in the model. A similar model may be constructed using the values at 1 week following initiation of anti-fungal therapy for each biomarker. In addition to the individual biomarker values, composite measures of the biomarkers over the 1st week and first 2 weeks of anti-fungal therapy such as the time weighted average, slope of straight line fitted to the data, time weighted average changes from baseline, and percent change from baseline may be used in separate logistic regression models.

The exploratory objective of examining the relationship between patient outcomes based on GMI and objective clinical outcome (i.e., survival vs. death) will be evaluated at 6 weeks and 12 weeks after initiation of anti-fungal therapy. To be included in this analysis, patients must be positive by GMI (GMI≥0.5) on at least 2 consecutive serum samples including the baseline serum sample. To be defined as persistently GMI negative (GMI<0.5) for at least 14 days after the first negative value, patients must have at least 2 valid GMI results (all of which must be negative) within 14 days following the first negative value. Kappa coefficient will be used to assess the correlation between outcome based on GMI and objective clinical outcome (i.e., survival vs. death).

3.5.6 Multiplicity

There is only 1 primary hypothesis for this study. Therefore no multiplicity adjustment is necessary. No multiplicity adjustment will be applied to the analysis of the secondary and exploratory endpoints. It is recognized that with so many analyses being performed, some may be statistically significant due to chance.

3.5.7 Sample Size and Power Calculations

The sample size for this study was calculated using information obtained from a retrospective analysis of a small subset of fungal biomarker data from CANCIDAS Protocols (MK0991 Protocols 019 and 037). Protocol 019 was an open, non-comparative trial in which Caspofungin therapy was given to patients with proven/probable invasive Aspergillus who were refractory to or intolerant of standard anti-Aspergillus therapy. Protocol 037 was an open, non-comparative trial in which Caspofungin therapy in combination with either a triazole or polyene was given to patients with proven/probable invasive Aspergillus who were refractory to or intolerant of standard anti-Aspergillus

therapy. Although these studies were conducted ~10 years ago, serum samples from patients in these studies had been banked and a subset were analyzed for the biomarkers (1,3)-β-D-glucan and galactomannan. Biomarker values and information on clinical outcome were available for 11 patients in Protocol 019 and 11 patients in Protocol 037. Within the first 2 weeks, 21 of the 22 patients had biomarker values for at least 4 time points while 1 patient had biomarker values for 2 time points. As mentioned in Section 3.5.5, for the primary hypothesis, for each patient, a time weighted average was calculated using the values obtained within the first 2 weeks (14 days) of the study for each biomarker. For each patient, a z-score was computed for the time weighted average for each biomarker by calculating the difference of the patient's time weighted average from the mean of the time weighted averages for the given biomarker and diving by the overall SD of the time weighted averages for the given biomarker. For each patient, O'Brien's global statistic was computed as the average of the z-scores of the time weighted averages across the biomarkers. The effect size was computed as the difference in the means of the average of the z-scores between the group of patients who experienced a clinical success (i.e., complete or partial cure) and the group of patients who experienced a clinical failure, divided by the pooled SD. The effect size was computed to be ~0.7. Similarly, using the methodology described in Section 3.5.5 for the secondary hypothesis, the effect size based on the difference in the means of the average of the z-scores of the slopes across the biomarkers between the group of patients who experienced a clinical success and those who experienced a clinical failure was computed to be ~0.5.

In this study, since patients are not being randomized to 2 groups in equal proportions, it is possible that there may be an imbalance between the proportion of patients who experience a clinical success and clinical failure in this study. In the Sims study, 88% of patients experienced a clinical success and the remaining 12% experienced a clinical failure [3]. In the previously mentioned retrospective analysis of a small subset of patients from MK0991 Protocols 019 and 037, 41% of patients experienced a clinical success and the remaining 59% experienced a clinical failure. Assuming that 40% of the patients will experience a clinical success (i.e., complete or partial recovery) and the remaining 60% will experience a clinical failure, with a total sample size of 50 patients and an assumed true effect size of 0.7 for the primary endpoint described above (i.e., average of the z-scores of the time weighted averages across the biomarkers), there is 78% power for the composite of the 2 biomarkers to discriminate between clinical success and clinical failure at the $\alpha = 0.05$ 1-sided level. Assuming that 50% of the patients will experience a clinical success and the remaining 50% of the patients will experience a clinical failure, with a total sample size of 50 patients and an assumed true effect size of 0.7, there is 80% power for the composite of the 2 biomarkers to discriminate between clinical success and clinical failure at the $\alpha = 0.05$ 1-sided level.

For the secondary hypothesis, assuming that 40% of the patients will experience a clinical success (i.e., complete or partial recovery) and the remaining 60% will experience a clinical failure, with a total sample size of 50 patients and an assumed true effect size of 0.5 for the secondary endpoint of the average of the z-scores of the slopes across the biomarkers, there is 54% power for the composite of the 2 biomarkers to discriminate between clinical success and clinical failure at the $\alpha = 0.05$ 1-sided level.

3.5.8 Subgroup Analyses and Effect of Baseline Factors

It is of interest to evaluate the relationship between the multiple fungal biomarkers and clinical outcome across various subgroups. Therefore, the primary analysis will be performed within the following subgroups: disease classification (probable or proven aspergillus infection), type of therapy (Amphotericin B, azoles, or echinocandins), co-existing/predisposing illness (neutropenia, immunosuppression therapy, or acquired immunodeficiency), patients receiving mold-active prophylaxis vs. those not receiving mold-active prophylaxis, and patients undergoing regular prospective biomarker screening vs. those not undergoing regular prospective biomarker screening.

3.6 CLINICAL SUPPLIES

3.6.1 Clinical Supplies Disclosure

This study is observational and uses standard-of-care anti-fungal therapy; therefore, the patient, the investigator's site personnel and the SPONSOR are not blinded to treatment. Disclosure envelopes are not provided.

3.7 DATA MANAGEMENT

Information regarding Data Management procedures for this protocol will be provided by the SPONSOR.

3.8 BIOLOGICAL SPECIMENS

Information regarding biological specimens for this protocol will be provided by the SPONSOR.

4. ADMINISTRATIVE AND REGULATORY DETAILS

4.1 CONFIDENTIALITY

4.1.1 Confidentiality of Data

For Studies Conducted Under the U.S. IND

Particular attention is drawn to the regulations promulgated by the Food and Drug Administration under the Freedom of Information Act providing, in part, that information furnished to clinical investigators and Institutional Review Boards will be kept confidential by the Food and Drug Administration only if maintained in confidence by the clinical investigator and Institutional Review Board.

For All Studies

By signing this protocol, the investigator affirms to the SPONSOR that information furnished to the investigator by the SPONSOR will be maintained in confidence and such information will be divulged to the Institutional Review Board, Ethics Review Committee, or similar or expert committee; affiliated institution; and employees only under an appropriate understanding of confidentiality with such board or committee, affiliated institution and employees. Data generated by this study will be considered confidential by the investigator, except to the extent that it is included in a publication as provided in the Publications section of this protocol.

4.1.2 Confidentiality of Subject/Patient Records

For All Studies

By signing this protocol, the investigator agrees that the SPONSOR (or SPONSOR representative), Institutional Review Board/Independent Ethics Committee (IRB/IEC), or Regulatory Agency representatives may consult and/or copy study documents in order to verify worksheet/case report form data. By signing the consent form, the subject/patient agrees to this process. If study documents will be photocopied during the process of verifying worksheet/case report form information, the subject/patient will be identified by unique code only; full names/initials will be masked prior to transmission to the SPONSOR.

For Studies Conducted Under the U.S. IND

By signing this protocol, the investigator agrees to treat all patient data used and disclosed in connection with this study in accordance with all applicable privacy laws, rules and regulations, including all applicable provisions of the Health Insurance Portability and Accountability Act and its implementing regulations, as amended from time to time. (“HIPAA”).

4.1.3 Confidentiality of Investigator Information

For All Studies

By signing this protocol, the investigator recognizes that certain personal identifying information with respect to the investigator, and all subinvestigators and study site

personnel, may be used and disclosed for study management purposes, as part of a regulatory submissions, and as required by law. This information may include:

- name, address, telephone number, and email address;
- hospital or clinic address and telephone number;
- curriculum vitae or other summary of qualifications and credentials; and
- other professional documentation.

Consistent with the purposes described above, this information may be transmitted to the SPONSOR, and subsidiaries, affiliates and agents of the SPONSOR, in your country and other countries, including countries that do not have laws protecting such information. Additionally, the investigator's name and business contact information may be included when reporting certain serious adverse events to regulatory agencies or to other investigators. By signing this protocol, the investigator expressly consents to these uses and disclosures.

For Multicenter Studies

In order to facilitate contact between investigators, the SPONSOR may share an investigator's name and contact information with other participating investigators upon request.

4.2 COMPLIANCE WITH LAW, AUDIT, AND DEBARMENT

By signing this protocol, the investigator agrees to conduct the study in an efficient and diligent manner and in conformance with this protocol; generally accepted standards of Good Clinical Practice; and all applicable federal, state, and local laws, rules and regulations relating to the conduct of the clinical study.

The Code of Conduct, a collection of goals and considerations that govern the ethical and scientific conduct of clinical investigations sponsored by Merck is attached.

The investigator also agrees to allow monitoring, audits, Institutional Review Board/Independent Ethics Committee review, and regulatory agency inspection of trial-related documents and procedures and provide for direct access to all study-related source data and documents.

The investigator agrees not to seek reimbursement from subjects/patients, their insurance providers, or from government programs for procedures included as part of the study reimbursed to the investigator by the SPONSOR.

The Investigator shall prepare and maintain complete and accurate study documentation in compliance with Good Clinical Practice standards and applicable federal, state, and local laws, rules and regulations; and, for each subject/patient participating in the study, provide all data, and upon completion or termination of the clinical study submit any

other reports to the SPONSOR as required by this protocol or as otherwise required pursuant to any agreement with the SPONSOR.

Study documentation will be promptly and fully disclosed to the SPONSOR by the investigator upon request and also shall be made available at the investigator's site upon request for inspection, copying, review, and audit at reasonable times by representatives of the SPONSOR or any regulatory agencies. The investigator agrees to promptly take any reasonable steps that are requested by the SPONSOR as a result of an audit to cure deficiencies in the study documentation and worksheets/case report forms.

International Conference of Harmonization Good Clinical Practice guidelines (Section 4.3.3) recommend that the investigator inform the subject's primary physician about the subject's participation in the trial if the subject has a primary physician and if the subject agrees to the primary physician being informed.

According to European legislation, a SPONSOR must designate a principal or coordinating investigator (CI) to review the report (summarizing the study results) and confirm that to the best of his/her knowledge the report accurately describes conduct and results of the study. The SPONSOR may consider one or more factors in the selection of the individual to serve as the CI (e.g., thorough understanding of clinical trial methods, appropriate enrollment of subject/patient cohort, timely achievement of study milestones, availability of the CI during the anticipated review process).

The investigator will promptly inform the SPONSOR of any regulatory agency inspection conducted for this study.

Persons debarred from conducting or working on clinical studies by any court or regulatory agency will not be allowed to conduct or work on this SPONSOR's studies. The investigator will immediately disclose in writing to the SPONSOR if any person who is involved in conducting the study is debarred, or if any proceeding for debarment is pending or, to the best of the investigator's knowledge, threatened.

In the event the SPONSOR prematurely terminates a particular trial site, the SPONSOR will promptly notify that site's IRB/IEC.

4.3 COMPLIANCE WITH FINANCIAL DISCLOSURE REQUIREMENTS

By signing this protocol, the investigator agrees to provide to the SPONSOR accurate financial information to allow the SPONSOR to submit complete and accurate certification and disclosure statements as required by U.S. Food and Drug Administration regulations (21 CFR Part 54). The investigator further agrees to provide this information on a Financial Disclosure/Certification Form that is provided by Merck Sharp & Dohme Corp., a subsidiary of Merck & Co., Inc. This requirement also extends to subinvestigators. The investigator also consents to the transmission of this information to Merck Sharp & Dohme Corp., a subsidiary of Merck & Co., Inc., in the United States for these purposes. This may involve the transmission of information to countries that do not have laws protecting personal data.

4.4 QUALITY CONTROL AND QUALITY ASSURANCE

By signing this protocol, the SPONSOR agrees to be responsible for implementing and maintaining quality control and quality assurance systems with written SOPs to ensure that trials are conducted and data are generated, documented, and reported in compliance with the protocol, accepted standards of Good Clinical Practice, and all applicable federal, state, and local laws, rules and regulations relating to the conduct of the clinical study.

4.5 COMPLIANCE WITH INFORMATION PROGRAM ON CLINICAL TRIALS FOR SERIOUS OR LIFE THREATENING CONDITIONS

Under the terms of The Food and Drug Administration Modernization Act (FDAMA), the SPONSOR of the study is solely responsible for determining whether the study is subject to the requirements for submission to the Clinical Trials Data Bank, http://clinicaltrials.gov/. Merck, as SPONSOR of this study, will review this protocol and submit the information necessary to fulfill this requirement. Merck entries are not limited to FDAMA mandated trials. Merck's voluntary listings, beyond those mandated by FDAMA, will be in the same format as for treatments for serious or life-threatening illnesses. Information posted will allow patients to identify potentially appropriate trials for their disease conditions and pursue participation by calling a central contact number for further information on appropriate study locations and site contact information.

By signing this protocol, the investigator acknowledges that the statutory obligation under FDAMA is that of the SPONSOR and agrees not to submit any information about this study to the Clinical Trials Data Bank.

4.6 PUBLICATIONS

As this study is part of a multicenter trial, publications derived from this study should include input from the investigator(s) and SPONSOR personnel. Such input should be reflected in publication authorship, and whenever possible, preliminary agreement regarding the strategy for order of authors' names should be established before conducting the study. Subsequent to the multicenter publication, or 24 months after completion of the study, whichever comes first, an investigator and/or his/her colleagues may publish the results for their study site independently. However, the SPONSOR does not recommend separate publication of individual study site results due to scientific concerns.

The SPONSOR must have the opportunity to review all proposed abstracts, manuscripts, or presentations regarding this study 60 days prior to submission for publication/presentation. Any information identified by the SPONSOR as confidential must be deleted prior to submission. SPONSOR review can be expedited to meet publication guidelines.

5. LIST OF REFERENCES

1. DePauw B. et al. Revised Definitions of Invasive Fungal Diseases from the European Organization for Research and Treatment of Cancer/Invasive Fungal Infections Cooperative Group and the National Institute of Allergy and Infectious Diseases Mycoses Study Group (EORTC/MSG) Consensus Group. Clin Infect Dis 2008;46: 1813-21.

2. Segal B.H., Herbrecht R., Stevens D.A., Ostrosky-Zeichner L., Sobel J., Viscoli C. et al. Defining Responses to Therapy and Study Outcomes in Clinical Trials of Invasive Fungal Infections: Mycoses Study Group (MSG) and European Organization for Research and Treatment of Cancer (EORTC) Consensus Criteria. [manuscript submitted to Clinical Infectious Diseases 2008.]

3. Sims C.R., Mohr J., Paetznick V., Rodriguez J., Finkelman M., Ostrosky-Zeichner L. Correlation of clinical outcomes with β-glucan (BG) levels in patients with invasive candidiasis. Program and abstracts of the 46th Interscience Conference on Antimicrobial Agents and Chemotherapy; September 27-30, 2006; San Francisco, California. Abstract M-1599.

4. Pfeiffer C.D., Fine J.P., Safdar N. Diagnosis of Invasive Aspergillosis Using a Galactomannan Assay: A Meta-Analysis. Clin Infect Dis 2006;42:1417-27.

5. Anaissie E.J. Trial Design for Mold-Active Agents: Time to Break the Mold—Aspergillosis in Neutropenic Adults. Clin Infect Dis 2007;44:1298-1306.

6. Pazos C., Ponton J., Del Palacio A. Contribution of (1→3)-β-D-Glucan Chromogenic Assay to Diagnosis and Therapeutic Monitoring of Invasive Aspergillosis in Neutropenic Adult Patients: a Comparison with Serial Screening for Circulating Galactomannan. J Clin Microbiol 2005;43(1):299-305.

7. Maertens J., Verhaegen J., Lagrou K., Van Eldere J., Boogaerts M. Screening for circulating galactomannan as a noninvasive diagnostic tool for invasive aspergillosis in prolonged neutropenic patients and stem cell transplantation recipients: a prospective validation. Blood 2001;97(6):1604-10.

8. Boutboul F. et al. Invasive Aspergillosis in Allogeneic Stem Cell Transplant Recipients: Increasing Antigenemia Is Associate with Progressive Disease. Clin Infect Dis 2002;34:939-43.

9. Woods G., Miceli M.H., Grazziutti M.L., Zhao W., Barlogie B., Anaissie E. Serum Aspergillus Galactomannan Antigen Values Strongly Correlate With Outcome of Invasive Aspergillosis: A Study of 56 Patients with Hematologic Cancer. Cancer 2007;110(4):830-4.

10. Miceli M.H. et al. Strong Correlation between Serum Aspergillus Galactomannan Index and Outcome of Aspergillosis in Patients with Hematological Cancer: Clinical and Research Implications. Clin Infect Dis 2008;46:1412-22.

11. Walsh T.J., Anaissie E.J., Denning D.W., Herbrecht R., Kontoyiannis D.P. et al. Treatment of Aspergillosis: Clinical Practice Guidelines of Infectious Diseases Society of America. Clin Infect Dis 2008; 46:327-60.

12. Stevens D.A. and Lee J.Y. Analysis of compassionate use itraconazole therapy for invasive aspergillosis by the NIAID Mycoses Study Group criteria. Arch Intern Med 1997;157:1857-62.

13. Loeffler J. et al. Nucleic Acid Sequence-Based Amplification of Aspergillus RNA in Blood Samples. J Clin Microbiol 2001;39(4):1626-9.

14. Yoo J.H. et al. Comparison of the Real-time Nucleic Acid Sequence-based Amplification (RTi-NASBA) with Conventional NASBA, and Galactomannan Assay for the Diagnosis of Invasive Aspergillosis. J Korean Med Sci 2007;22:672-6.

15. Yoo J.H. et al. Application of Nucleic Acid Sequence-based Amplification for Diagnosis of and Monitoring the Clinical Course of Invasive Aspergillosis in Patients with Hematologic Diseases. Clin Infect Dis 2005;40:392-8.

16. Perlin, D.S. and Y. Zhao, Molecular diagnostic platforms for detecting Aspergillus. Med Mycol, 2009. 47 Suppl 1: p. S223-32.

17. Zhao, Y., et al., Rapid real-time nucleic Acid sequence-based amplification-molecular beacon platform to detect fungal and bacterial bloodstream infections. J Clin Microbiol, 2009. 47(7): p. 2067-78.
